# Associations between dietary mycotoxins exposures and risk of hepatocellular carcinoma in a European cohort

**DOI:** 10.1371/journal.pone.0315561

**Published:** 2024-12-16

**Authors:** Inge Huybrechts, Inarie Jacobs, Carine Biessy, Elom K. Aglago, Mazda Jenab, Liesel Claeys, Jiri Zavadil, Corinne Casagrande, Genevieve Nicolas, Ghislaine Scelo, Andrea Altieri, Beatrice Fervers, Isabelle P. Oswald, Julien Vignard, Bernadette Chimera, Maria Santucci de Magistris, Giovanna Masala, Domenico Palli, Lisa Padroni, Jesús Castilla, Ana Jiménez-Zabala, Pauline Frenoy, Francesca Romana Mancini, Xuan Ren, Emily Sonestedt, Paolo Vineis, Alicia Heath, Mårten Werner, Esther Molina-Montes, Christina C. Dahm, Fie Langmann, José María Huerta, Magritt Brustad, Guri Skeie, Matthias B. Schulze, Antonio Agudo, Sabina Sieri, Michael Korenjak, Marc J. Gunter, Sarah De Saeger, Marthe De Boevre

**Affiliations:** 1 International Agency for Research on Cancer (IARC/WHO), Nutrition and Metabolism Branch, Lyon, France; 2 CRIG, Cancer Research Institute Ghent, Ghent, Belgium; 3 International Agency for Research on Cancer (IARC/WHO), Epigenomics and Mechanisms Branch, Lyon, France; 4 European Food Safety Authority (EFSA), Parma, Italy; 5 Centre Léon Bérard, Lyon, France; 6 Toxalim (Research Centre in Food Toxicology), INRAE, ENVT, INP-Purpan, UPS, Université de Toulouse, Toulouse, France; 7 Azienda Ospedaliera Universitaria (AOU) Federico II, Naples, Italy; 8 Institute for Cancer Research, Prevention and Clinical Network (ISPRO), Florence, Italy; 9 Unit of Cancer Epidemiology, Città della Salute e della Scienza University-Hospital and Center for Cancer Prevention (CPO), Turin, Italy; 10 Instituto de Salud Pública de Navarra–IdiSNA, Pamplona, Spain; 11 CIBER of Epidemiology and Public Health (CIBERESP), Madrid, Spain; 12 Ministry of Health of the Basque Government, Sub Directorate for Public Health and Addictions of Gipuzkoa, San Sebastian, Spain; 13 BioGipuzkoa Health Research Institute, Epidemiology of Chronic and Communicable Diseases Group, San Sebastián, Spain; 14 UVSQ, Inserm "Exposome, Heredity, Cancer and Health" Team, CESP U1018, Gustave Roussy, Université Paris-Saclay, Villejuif, France; 15 Department of Clinical Sciences Malmö, Lund University, Malmö, Sweden; 16 Cancer Epidemiology and Prevention Research Unit, School of Public Health, Imperial College London, London, United Kingdom; 17 Department of Public Health and Clinikal Medicine, Umeå University, Umeå, Sweden; 18 Department of Nutrition and Food Science, Campus of Cartuja, University of Granada, Granada, Spain; 19 Instituto de Investigación Biosanitaria ibs.GRANADA, Granada, Spain; 20 Institute of Nutrition and Food Technology (INYTA) ‘José Mataix’, Biomedical Research Centre, University of Granada, Granada, Spain; 21 Dept. of Public Health, Aarhus University, Aarhus, Denmark; 22 Department of Epidemiology, Murcia Regional Health Council-IMIB, Murcia, Spain; 23 Department of Community Medicine, Faculty of Health Sciences, UiT The Arctic University of Norway, Tromsø, Norway; 24 The Public Dental Health Service Competence Centre of Northern Norway, Tromsø, Norway; 25 Department of Molecular Epidemiology, German Institute of Human Nutrition Potsdam-Rehbruecke, Nuthetal, Germany; 26 Institute of Nutritional Science, University of Potsdam, Nuthetal, Germany; 27 Unit of Nutrition and Cancer, Catalan Institute of Oncology ‐ ICO, L’Hospitalet de Llobregat, Spain; 28 Nutrition and Cancer Group, Bellvitge Biomedical Research Institute ‐ IDIBELL, L’Hospitalet de Llobregat, Spain; 29 Epidemiology and Prevention Unit, Fondazione IRCCS Istituto Nazionale dei Tumori, Milan, Italy; 30 Centre of Excellence in Mycotoxicology and Public Health, Ghent University, Ghent, Belgium; Ladoke Akintola University of Technology, NIGERIA

## Abstract

Mycotoxins have been hypothesized to contribute to a diversity of adverse health effects in humans, even at low concentrations. Certain mycotoxins are established human carcinogens, whereas for others research suggests potential carcinogenic effects. The aim of this study was to determine the association between dietary exposure to mycotoxins and hepatobiliary cancers in the European Prospective Investigation into Cancer and Nutrition (EPIC) cohort. EPIC questionnaire data were matched to mycotoxin food occurrence data compiled by the European Food Safety Authority to assess long-term dietary mycotoxin exposure (expressed as μg/kg body weight/day) and then relate them to the risk of hepatocellular carcinoma (HCC) (n = 255) and biliary tract cancers (n = 273). Analyses were conducted using multivariable Cox proportional hazards regression models to compute hazard ratios (HR) and 95% confidence intervals (95% CI). Key food groups contributing to mycotoxin exposure were cereals and cereal-based products, vegetables, non-alcoholic beverages (including fruit juices) and fruits. Estimated intake of deoxynivalenol (DON) and its derivatives was positively associated with HCC risk (HR_T3vsT1_: 1.90, 95% CI: 1.18–3.05, *p-*trend <0.01). No statistically significant associations were found for the other mycotoxins. Further research to confirm our observations and investigate potential underlying mechanisms of these compounds is warranted. These data may provide evidence of HCC risks associated with higher dietary intake levels of DON, which has not yet been classified as a human carcinogen.

## Introduction

Liver cancer is the sixth most frequently diagnosed cancer worldwide with an estimated 1 053 619 new cases in 2030 and an age-standardized incidence rate of 8.6 per 100,000 person-years [1]. Liver cancer is one of the deadliest cancers, with an age-standardized annual mortality rate of 7.4 per 100,000 person-years [1]. Liver cancer incidence and mortality continue to rise, despite advances in prevention strategies and new technologies in both diagnosis and treatment [2]. Liver cirrhosis is the most important risk factor for the development of hepatocellular carcinoma (HCC, which comprise the majority of liver cancers), with hepatitis B and C and alcohol consumption, unhealthy dietary patterns, smoking and obesity being among other major risk factors for the development of liver cirrhosis [3].

Mycotoxins are fungal secondary metabolites that contaminate many of the most frequently consumed foods worldwide, such as grains, nuts, fruits and legumes [4–6]. One fungal species may produce many different mycotoxins, and the same mycotoxin may also be produced by several different fungal species [5, 6]. Recent reports revealed that 60% to 80% of the world’s food crops are contaminated by mycotoxins, resulting in a widespread human exposure to one or more mycotoxins, through consumption of cereal-based foods, nuts, fruits, coffee, spices, and oil-based seeds [7–9]. Chronic exposure to mycotoxins, such as aflatoxins (AFs), have been classified as potent human hepatocarcinogens [10–12].

Numerous mycotoxins have been identified but the mycotoxins most commonly related to human health include AFs, ochratoxin A, patulin, fumonisins, zearalenone and nivalenol/deoxynivalenol (see S1 Table) [13]. The International Agency for Research on Cancer (IARC) identifies the individual AFs B1 (AFB1), B2 (AFB2), G1 (AFG1), G2 (AFG2) and M1 (AFM1) as sufficiently evident human carcinogens, while other mycotoxins are designated as possible carcinogens (*e*.*g*., ochratoxin A (OTA), fumonisin B1 (FB1) fumonisin B2 (FB2). Recent research indicates that exposure to multiple mycotoxins have a potentially increased carcinogenic effect over single mycotoxins [14–17].

The primary target organ for many mycotoxins is the liver, evidenced especially by their association with HCC [18], where they are metabolized, though not always inactivated [19–21]. There is a strong, established link between AFs exposure and development of HCC [12, 17, 21]. The World Cancer Research Fund (WCRF) has further concluded strong evidence linking AF-contaminated foods with liver cancer risk [22]. The associated mechanism possibly involves metabolization of AFB1 in the liver to a highly reactive species, capable of forming mutagenic DNA-adducts. Subsequently, a synergistic effect promoting tumour growth in the liver has been reported for co-exposure to AFB1 and FB1 [23]. Less pronounced effects were reported in a meta-analysis on co-exposure to OTA, also resulting in increased hepatic lipid levels and increased relative weight of the organ [4].

Recent reports identify various food safety issues such as the presence of mycotoxins, likely to be affected by climate change, particularly in Europe. Fungal shifts towards northern latitudes due to warmer conditions give rise to a subsequent emerging pattern of mycotoxins in different regions in Europe [24–27]. Given the ubiquitous nature of exposures to multiple mycotoxins in many countries, it is imperative to comprehensively investigate their hepatocarcinogenic potency, by means of longitudinal cohort data. Therefore, this manuscript determines the association between mycotoxin exposures and incidence of HCC cancer using a large-scale multinational European cohort study.

## Materials and methods

### Subjects and study design

The European Prospective Investigation into Cancer and Nutrition (EPIC) study is a large on-going multicentre prospective cohort study consisting of 521,323 adults (367,898 women and 153,425 men) mostly recruited aged 35–70 years from whom diet, and lifestyle data were collected at baseline. The participants were enrolled between 1992 and 2000 from 23 centres in 10 European countries: Denmark, France, Germany, Italy, The Netherlands, Norway, Spain, Greece, Sweden and the United Kingdom [28]. The rationale, study population and data collection have been described elsewhere [29]. All participants provided written informed consent and the ethical review boards from IARC and from all local centres approved the study. Participants with prevalent cancer at baseline (n = 25,184), and with missing information on lifestyle or dietary information (n = 6,259), follow-up information (n = 4,148), or in the highest and lowest distribution percentiles for the ratio energy intake to estimated energy requirement (n = 9,573) were excluded from the analysis. Data from Greek participants (n = 26,048) were not available. The final study population included 450,112 participants (70% females).

### Dietary data and lifestyle questionnaires

#### Dietary questionnaires

Usual dietary intake was assessed at study baseline using validated country or centre-specific dietary questionnaires (DQs) [29]. In most centres, DQs were self-administered, except for Ragusa (Italy), Naples (Italy), and Spain, where face-to-face interviews were performed. Semi-quantitative DQs were used in Italy, the Netherlands and Denmark, while diet history questionnaires were structured by meals in Spain and France. Food-frequency questionnaires (FFQs) were used in Germany, the United Kingdom, and Umeå (Sweden). A method combining a short FFQ with a 7-day record on hot meals was used in Malmö (Sweden). The nutritional values of consumed foods were taken from the EPIC Nutrient Database (ENDB), compiled using a highly standardised procedure, adopting nutrient values from the national food composition databases of the respective EPIC countries. The process for compiling this ENDB database has been previously described [30]. Information on physical activity, history of tobacco smoking, alcohol consumption and education were collected at baseline by questionnaires. Weight and height were measured at the baseline examination in all centres except from part of Oxford, France and Norway, where weight and height were self-reported [28].

#### Mycotoxin occurrence data

The European Food Safety Authority (EFSA) is an agency of the European Union (EU), tasked with performing risk assessments regarding safety of foods for human consumption and feeds for livestock. The EFSA databases relevant to this project record mycotoxin occurrences of all types of mycotoxins, filed in Europe and obtained via the European members states. EFSA provides guidance to report analysed data, when launching calls for data to the member states. For this study, mycotoxin occurrence data derived from the EFSA database was used and matched with the EPIC food consumption data derived from the dietary questionnaires. Mycotoxin contents of foods analysed from 1998 to 2013, as provided by EFSA, were used for these analyses. To calculate the quantity of each mycotoxin consumed by a specific individual, the portion (in grams) of every food that was consumed by each participant (as reported in the DQs) was linked to the mycotoxin occurrence data (196 321 food samples analysed overall) for that particular food [9]. Unfortunately, the data did not allow country-specific matching between the dietary intake data and the food occurrence data [9].

#### Mycotoxin concentration scenarios assigned to non-detect samples

When reporting contaminant concentrations analysed in monitoring programmes, actual numeric values of concentrations are only reported when the measurements exceed the limit of detection (LOD) or limit of quantification (LOQ). If less than these limits, samples are classified as non-detect samples. To calculate exposures for these non-detect samples, a medium bound (MB)-concentration scenario was applied in which all non-detected samples of commodities (including drinking water) with at least one sample with a concentration at or above the LOD or LOQ were assigned a concentration equal to half the limit value. The remaining non-detect samples were assumed to contain no mycotoxins. This scenario was chosen as opposed to assigning all non-detect samples a concentration equal to 0 μg/kg (so called lower bound scenario) to link the analysed concentrations to the foods consumed. However, also the lower bound scenario was used in this study for conducting sensitivity analyses.

#### Mycotoxin grouping for analysis

Groups of related mycotoxins were determined according to certain families depending on their chemical structure. Concentration levels were computed by summing the levels of mycotoxins in the group. The group *Aflatoxins* included AFB1, AFB2, AFG1, AFG2, AFM1. The group *Deoxynivalenol (DON) and derivatives* included DON, 3-acetyl-DON (3-ADON), 15-acetyl-DON (15-ADON) and deoxynivalenol-3-glucoside (DON3G). The *Fumonisins* group included fumonisin B1 (FB1), fumonisin B2 (FB2) and fumonisin B3 (FB3). *Zearalenone and derivatives* were compiled by the zearalenone (ZEN), zearalenone-derivatives (ZEN-dv), zearalenols (ZEL) includingα-zearalenol (A_ZEL) and β-zearalenol (B_ZEL), and zearalanone (ZAN). The group *Alternaria toxins* included alternariol (AOH), alternariol mono-methylether (AME), altenuene (ALT), tenuazonic acid (TEA), altertoxin (ATX), tenuazonic acid (TEN) and AAL-toxins (AAL_toxins). The group *Enniatins* included enniatin A (ENN_A), enniatin A1 (ENN_A1), enniatin B (ENN_B) and enniatin B1 (ENN_B1). The group *Ergot alkaloids* included ergocornine (Eco), ergocorninine (Econ), ergocristine (Ecr), ergocristinine (Ecrn), α-ergokryptine (Ek), α-ergokryptinine (Ekn), ergometrine (Em), ergometrinine (Emn), ergosine (Es), ergosinine (Esn), ergotamine (Et) and ergotaminine (Etn). The group *Ochratoxins* included OTA. The group *T2 & HT2* included HT-2 toxin (HT2) and T-2 toxin (T2). Other mycotoxins were handled individually: patulin (PAT), nivalenol (NIV), diacetoxyscirpenol (DAS), fusarenon-X (FUS-X), moniliformin (MON), citrinin (CIT), beauvericin (BEAU) and sterigmatocystin (STC) (see S2 Table).

#### Follow-up for cancer incidence and vital status

Incident cancer cases were identified through several methods, including record linkage with population-based cancer registries, health insurance records, pathology registries, and active follow-up of study participants and their close kinship. Data on vital status were obtained from mortality registries, in combination with data collected through active follow-up in some of the centres. Exit time was the age at whichever of the following came first: liver cancer diagnosis, death, or the last date at which follow-up was considered complete.

For HCC, cases were defined as first incident primary cancer and were coded according to the International Classification of Diseases for Oncology (ICD-O) [31]. HCC was defined as C22.0 with morphology codes ICD-O-2 “8170/3”, 8171/3 and “8180/3” and IHBC as C22.1 (all morphology codes except ICD-O-2 “8162/3” which was recoded as extrahepatic bile duct). For each identified HCC case, the histology, and the methods, used to diagnose the cancer, were reviewed by a pathologist to exclude metastatic cases or other types of primary liver cancers. In addition (as part of secondary objective), malignant neoplasm of gallbladder (C23) and malignant neoplasm of other and unspecified parts of biliary tract (C24) were included. Our definition of gallbladder and biliary tract cancers (GBTC) includes tumours in the gallbladder (C23.9 (morphology codes: 8000/3, 8010/2, 8010/3, 8020/3, 8140/3, 8160/3, 8260/3, 8480/3, 8490/3, 8560/3)), extrahepatic bile ducts (C24.0 (morphology codes: 8000/3, 8010/3, 8140/3, 8160/3, 8162/3, 8260/3)), ampulla of Vater (C24.1 (morphology codes: 8000/3, 8010/3, 8140/3, 8260/3, 8480/3, 8481/3, 8490/3)), and overlapping (C24.8 (morphology codes: 8000/3, 8160/3) and non-specified tumors of the biliary tract (C24.9 (morphology codes: 8000/3, 8001/3, 8010/3, 8140/3, 8160/3, 8162/3, 8480/3, 8481/3).

#### Statistical analysis

Analyses of the association between dietary multi-mycotoxin exposures with hepatobiliary cancer risk were conducted by performing Cox proportional hazards regression estimating hazard ratios (HRs) and 95% confidence intervals (CIs). Tertiles of each mycotoxin were used to calculate the associated hazard ratios. Time at risk was estimated from the time of recruitment to the time of death, emigration, loss to follow-up, or the end of follow-up period (a maximum through 2014 depending on centre), whichever occurred first. To control for differences in questionnaire design and follow-up procedures, models were further stratified by age at recruitment (one-year category) and sex. The entry time was defined as age at recruitment, while exit time was age occurrence of the event (*i*.*e*., age at last follow-up, first diagnosis of incident cancer, loss to follow-up, or death, whichever came first). Trend tests across levels of exposure were performed on standardized continuous variables (association per 1 SD increase), while for categorical variables the test has been computed by assigning consecutive scores to the categories (sex-specific tertiles) as an ordinal variable (1 to 3). Finally, confounding factors remained in the models if the *β*-estimate changed by more than 10%. On the basis of these conditions, common adjustments factors included highest level of attained education (none or primary school completed, technical/professional school or secondary school, longer education, not specified), body mass index (BMI continuous), total energy intake (continuous in Kcal/day), coffee consumption (continuous in g/day), alcohol intake at recruitment (continuous in g/day) and lifetime alcohol intake (continuous in g/day), smoking status (never, former, current, missing), self-reported diabetes (type 1 and 2) at baseline (yes, no, missing), and physical activity (inactive or moderately inactive, moderately active or active, missing).

Analyses were based on mycotoxin intakes (in μg) divided by kilos of body weight (most common mode of expression). Although our main objective was to investigate associations between the individual mycotoxins and HCC, some extra explorative analyses were run, investigating potential effects of total multi-mycotoxins exposures and with groups of mycotoxins by summing the levels of mycotoxins belonging to certain families depending on their physicochemical properties. We computed the trend on the standardized continuous variable as the interpretation is easier and comparable across the different mycotoxins for which the levels can be very different.

For sensitivity analyses, models were fitted for men and women, separately. Multivariable models were adjusted for known or suspected risk factors, sex, age, and study centre for each liver cancer based on the findings of the World Cancer Research Fund/American Institute for Cancer Research [22]. Additionally, sensitivity analyses were conducted without adjustment for coffee consumption and extra analyses were run in a sub-cohort in which information on hepatitis infection status was available from case-control studies nested in EPIC, allowing extra adjustment for hepatitis infection.

Statistical analyses were performed with the SAS, version 9.4 statistical software package. All tests of statistical significance were two-sided and P-values below 0.01 were considered significant (after adjusting for multiple testing). The association between mycotoxins and HCC was explored in multivariate conditional regression models using the Benjamini-Hochberg correction to control for multiple comparisons [32].

## Results and discussion

Descriptive analyses indicated differences between the hepatobiliary cancer cases (N = 255) and the non-cases for age at recruitment, BMI, alcohol consumption, sex, smoking status, energy intake and the percentage classified as physically inactive and with diagnosed diabetes (Table 1).

**Table 1:** Characteristics of the EPIC study participants.

Total number of samples after exclusions = 450,112	HCC Cases		Non-HCC cases	
	N = 255		N = 449,857	
	Mean	SD	Mean	SD
Body Mass Index (kg/m^2^)	28.3	5.1	25.3	4.2
Age at recruitment (years)	58.2	6.9	51.1	9.8
Energy intake USDA (kcal)	2270.9	702.2	2076.4	618.7
Alcohol intake at recruitment (g/d)	23.4	35.4	11.7	16.8
Length of follow-up (years)	9.6	4.9	14.1	3.9
	**n**	**%**	**n**	**%**
**Sex**				
Male	162	63.5	131 264	29.2
Female	93	36.5	318 593	70.8
**Education**				
None	10	3.9	15 541	3.5
Primary school completed	104	40.8	110 960	24.7
Technical/professional school	65	25.5	103 718	23.1
Secondary school	25	9.8	93 885	20.9
Longer education (incl. University deg.)	44	17.3	108 887	24.2
Missing	7	2.7	168 66	3.7
**Physical activity**				
Inactive			87 950	19.6
Moderately inactive	82	32.2	149 867	33.3
Moderately active	74	29	120 153	26.7
Active	46	18	83 063	18.5
Missing	53	20.8	8 824	2.0
**Diabetes**				
No	195	76.5	400 257	89.0
Yes	31	12.2	10 707	2.4
Missing	29	11.4	38 893	8.6
**Smoking status**				
Never	75	29.4	219 219	48.7
Former	79	31	122 601	27.3
Smoker	99	38.8	99 616	22.1
Missing	2	0.8	8 421	1.9
Hepatitis status*				
No	69	67.7	700	94.9
Yes	33	32.3	38	5.1

HCC: hepatocellular carcinoma

A large part of the EPIC population is being exposed to some of the main mycotoxins present in foods, although for most of the mycotoxins, only a small percentage of the population has exposures above the Tolerable Daily Intake (TDI) (Table 2). For mycotoxins like AFs and STC for which a tolerable intake of 0 μg/kg body weight (*cf*. as low as reasonably achievable (ALARA)-principle) is considered, nearly the whole population showed an intake above this 0-value leading to almost 100% of the population with intakes higher than the TDI in the MB scenario (Table 2B). For all other mycotoxins the percentage of the population that had intakes above the TDI (according to middle bound values) was rather low (ranging from 0% for FB up close to 3% for DAS) in the MB scenario (Table 2B).

**Table 2:** Percentage of the population below and above the safety reference values for external mycotoxin exposures assessed based upon dietary questionnaire data for the full EPIC cohort (Table 2A: lower bound values; Table 2B: middle bound values).

(A) Lower Bound (LB) - μg/kg body weight						
LABEL (expressed in -μg/kg body weight)	Type	Cut-off	N < = cut-off	%	N > cut-off	%
Ergot alkaloids LB [33]	TDI	0.6	476,760	99.998	9	0.002
Ochratoxins sum LB [34]	TDI	0.016	476,637	99.972	132	0.028
Ochratoxins sum LB [34]	**PTWI**	0.112	476,769	100	0	0
Ochratoxins sum LB [34]	**TWI**	0.1714	476,769	100	0	0
Aflatoxin sum LB [35]	TDI	0	7,459	1.564	469,310	98.436
Aflatoxin sum LB [35]	**BMDL**	0.17	476,769	100	0	0
Patulin LB [36]	TDI	0.4	476,769	100	0	0
Deoxynivalenol & deriv. LB [37]	**PMTDI**	1	476,757	99.997	12	0.003
T-2/HT-2 toxins sum LB [38]	TDI	0.1	476,769	100	0	0
Nivalenol LB [39]	TDI	1.2	476,769	100	0	0
Nivalenol LB [39]	TDI	0.7	476,769	100	0	0
Fumonisins sum LB [40]	TDI	2	476,766	99.999	3	0.001
*Diacetoxyscirpenol LB [*41]	*TDI*	*0*.*06*	*476*,*769*	*100*	*0*	*0*
Zearalenone & deriv. SUM LB [42]	TDI	0.25	476,557	99.956	212	0.044
*Sterigmatocystin LB* [43]	*TDI*	*0*	*476*,*769*	*100*	* 0*	* 0*
**(B) Middle Bound (MB) - μg/kg body weight**						
**LABEL (expressed in -μg/kg body weight)**	**Type**	**Cut-off**	**N < = cut-off**	**%**	**N > cut-off**	**%**
Ergot alkaloids MB [33]	TDI	0.6	476,658	99.977	111	0.023
Ergot alkaloids MB [33]	**ARfD**	1	476,764	99.999	5	0.001
Ochratoxins sum MB [34]	TDI	0.016	476,576	99.96	193	0.04
Ochratoxins sum MB [34]	**PTWI**	0.112	476,769	100	0	0
Ochratoxins sum MB [34]	**TWI**	0.1714	476,769	100	0	0
Aflatoxin sum MB [35]	TDI	0	0	0	476,769	100
Aflatoxin sum MB [35]	**BMDL**	0.17	476,769	100	0	0
Patulin MB [36]	TDI	0.4	476,769	100	0	0
Deoxynivalenol & deriv. MB [37]	**PMTDI**	1	476,422	99.927	347	0.073
T-2/HT-2 toxins sum MB [38]	TDI	0.1	476,759	99.998	10	0.002
Nivalenol MB [39]	TDI	1.2	476,769	100	0	0
Nivalenol MB [39]	TDI	0.7	476,769	100	0	0
Fumonisins sum MB [40]	TDI	2	476,766	99.999	3	0.001
*Diacetoxyscirpenol MB* [*41*]	*TDI*	*0*.*06*	*463*,*493*	*97*.*215*	*13*,*276*	*2*.*785*
Zearalenone & deriv. SUM MB [42]	TDI	0.25	476,385	99.919	384	0.081
*Sterigmatocystin MB* [43]	*TDI*	*0*	*1*,*707*	*0*.*358*	*475*,*062*	*99*.*642*

TDI; tolerable daily intake, TWI; tolerable weekly intake, PMTDI, provisional maximum tolerable daily intake, PTWI; provisional tolerable weekly intake

Mycotoxins for which only insignificant values have been detected are written in Italic font (Diacetoxyscirpenol, Sterigmatocystin).

S3–S6 Tables presents a description of the external mycotoxin exposures assessed based upon dietary questionnaire data for the full EPIC cohort for lower bound values and middle bound value for μg/d and μg/kg body weight/d. For some of the mycotoxins such as citrinin, only few insignificant values had been measured/detected in the foods that were analysed by the member states (see lower bound scenario equal to zero for all percentiles). However, low values were assigned when the measurement was below the LOD or LOQ in the middle-bound scenario where half of the LOD or LOQ was considered for the MB scenario.

Associations between mycotoxin exposures and liver cancer risk were investigated and have shown an increased risk for DON with HCC, which remained significant in the fully adjusted model (HR_T3vsT1_ (95%CI) = 1.90 (1.18–3.05), p-trend = 0.0079) (Table 3). It is noteworthy that an inverse association was found for MON and HCC (HR_T3vsT1_ (95%CI) = 0.65 (0.43–0.99), p-trend = 0.042) and Citrinin and HCC (HR_T3vsT1_ (95%CI) = 0.59 (0.39–0.89), p-trend = 0.013. No significant risks were found for the other mycotoxins in the fully adjusted model. Sensitivity analysis in which we additionally adjust for hepatitis infection in a subsample of the cohort for which hepatitis infection status was available confirmed these statistically significant associations for DON (HR_T3vsT1_ (95%CI) = 2.25 (1.11–4.57)) while no significant associations were found for the other mycotoxins (although same positive trend was found, results were attenuated due to reduced power and limited number of cases with hepatitis and should therefore be interpreted with caution; S7 Table). Results for the model that does not include coffee consumption as potential confounding factor were similar to those of the fully adjusted model and are included as S8 Table. Sensitivity analysis showed that results obtained in Table 3 did not differ between male and female participants (although same positive trend was found, this was attenuated for women due to reduced power and limited number of cases for women and should therefore be interpreted with caution; S9 Table). Additional analysis was performed to investigate the associations between mycotoxin exposure and intrahepatic biliary tract cancer risk, extra-hepatic biliary tract cancer risk and gall bladder and biliary tract cancer risk. No significant risks were found in this analysis (S10–S12 Tables).

**Table 3:** Hazard ratios (HR) and their 95% confidence intervals (CI) for the associations between mycotoxin (body weight) exposures and liver cancer risk using a fully adjusted model*. P-value of 0.01 was considered statistically significant (after Bonferroni correction).

	MB (middle bound)	HCC (body weight)				
	Mycotoxins μg/BW/day	Cases 255	HR	95% CI	Probability Chi Square	Ptrend
Ergot alkaloids	Per 1 SD increase		1.03	0.87–1.23	0.7253	.
	Τ1	72	1	Ref.	.	.
	Τ2	96	0.99	0.63–1.56	0.9769	0.6304
	Τ3	87	1.12	0.66–1.91	0.6674	.
Ochratoxins	Per 1 SD increase		1.01	0.82–1.25	0.9078	.
	Τ1	105	1	Ref.	.	.
	Τ2	79	0.88	0.61–1.26	0.4762	0.6568
	Τ3	71	0.91	0.59–1.41	0.6765	.
Aflatoxins	Per 1 SD increase		0.97	0.77–1.23	0.8064	.
	Τ1	114	1	Ref.	.	.
	Τ2	85	0.93	0.64–1.33	0.6750	0.5640
	Τ3	56	0.88	0.55–1.39	0.5721	.
Patulin	Per 1 SD increase		1.28	1.16–1.41	< .0001	.
	Τ1	82	1	Ref.	.	.
	Τ2	78	0.92	0.63–1.35	0.6814	0.1340
	Τ3	95	1.32	0.91–1.92	0.1440	.
Deoxynivalenol and derivatives	Per 1 SD increase		1.12	0.96–1.30	0.1600	.
	Τ1	85	1	Ref.	.	.
	Τ2	74	1.22	0.81–1.82	0.3389	**0.0079**
	Τ3	96	**1.9**	**1.18–3.05**	**0.0084**	**.**
T-2/HT-2 toxins	Per 1 SD increase		1.20	1.02–1.40	0.0249	.
	Τ1	84	1	Ref.	.	.
	Τ2	68	0.84	0.57–1.25	0.3955	0.1813
	Τ3	103	1.28	0.86–1.91	0.2240	.
Nivalenol	Per 1 SD increase		1.04	0.86–1.25	0.6836	.
	Τ1	88	1	Ref.	.	.
	Τ2	77	0.87	0.58–1.30	0.4881	0.2349
	Τ3	90	1.31	0.83–2.07	0.2444	.
Fumonisins	Per 1 SD increase		1.07	0.89–1.29	0.4436	.
	Τ1	97	1	Ref.	.	.
	Τ2	74	0.97	0.66–1.43	0.8784	0.4668
	Τ3	84	1.18	0.75–1.84	0.4710	.
*Diacetoxyscirpenol*	Per 1 SD increase		*0*.*98*	*0*.*86–1*.*12*	*0*.*7729*	.
	* Τ1*	*109*	*1*	*Ref*.	.	.
	* Τ2*	*76*	*0*.*97*	*0*.*67–1*.*42*	*0*.*8905*	*0*.*9254*
	* Τ3*	*70*	*0*.*98*	*0*.*61–1*.*56*	*0*.*9323*	.
Zearalenone & derivatives	Per 1 SD increase		1.01	0.83–1.24	0.9026	.
	Τ1	104	1	Ref.	.	.
	Τ2	81	0.92	0.62–1.34	0.6495	0.2590
	Τ3	70	0.76	0.47–1.22	0.2544	.
Fusarium Toxins	Per 1 SD increase		1.11	0.93–1.33	0.2424	.
	Τ1	89	1	Ref.	.	.
	Τ2	80	1.21	0.82–1.80	0.3310	0.1148
	Τ3	86	1.46	0.91–2.35	0.1150	.
*Fusarenon X*	Per 1 SD increase		1.18	0.97–1.42	*0*.*0958*	.
	* Τ1*	*80*	*1*	*Ref*.	.	.
	* Τ2*	*79*	*1*.*08*	*0*.*73–1*.*59*	*0*.*7111*	*0*.*1069*
	* Τ3*	*96*	*1*.*45*	*0*.*92–2*.*28*	*0*.*1083*	.
*Sterigmatocystin*	Per 1 SD increase		*0*.*79*	*0*.*55–1*.*13*	*0*.*1941*	.
	* Τ1*	*111*	*1*	*Ref*.	.	.
	* Τ2*	*84*	*1*.*02*	*0*.*73–1*.*41*	*0*.*9092*	*0*.*1070*
	* Τ3*	*60*	*0*.*65*	*0*.*42–1*.*02*	*0*.*0621*	.
Moniliformine	Per 1 SD increase		0.98	0.80–1.20	0.8427	.
	Τ1	102	1	Ref.	.	.
	Τ2	81	0.82	0.58–1.17	0.2767	0.0429
	Τ3	72	0.65	0.43–0.99	0.0451	.
Alternaria toxins	Per 1 unit increase		1.17	0.94–1.45	0.1640	.
	Τ1	69	1	Ref.	.	.
	Τ2	98	1.52	1.02–2.28	0.0398	0.0826
	Τ3	88	1.58	0.95–2.62	0.0755	.
*Citrinin*	Per 1 SD increase		*0*.*83*	*0*.*67–1*.*02*	*0*.*0758*	.
	* Τ1*	*99*	*1*	*Ref*.	.	.
	* Τ2*	*86*	*0*.*89*	*0*.*63–1*.*26*	*0*.*5201*	*0*.*0138*
	* Τ3*	*70*	*0*.*59*	*0*.*39–0*.*89*	*0*.*0121*	.
Enniatins	Per 1 SD increase		0.99	0.78–1.26	0.9433	.
	Τ1	83	1	Ref.	.	.
	Τ2	80	0.98	0.67–1.45	0.9336	0.8460
	Τ3	92	1.07	0.62–1.83	0.8167	.
Sum of Mycotoxins	Per 1 SD increase		1.16	0.95–1.43	0.1465	.
	Τ1	78	1	Ref.	.	.
	Τ2	87	1.41	0.94–2.11	0.0953	0.0501
	Τ3	90	1.66	1.00–2.74	0.0488	.
Sum of Mycotoxins, using z-scores	Per 1 SD increase		1.06	0.85–1.32	0.5892	.
	Τ1	86	1	Ref.	.	.
	Τ2	86	1.10	0.75–1.63	0.6191	0.5196
	Τ3	83	1.18	0.72–1.93	0.5207	.

T1; Tertile 1, T2; Tertile 2, T3; Tertile 3, HCC; Hepatocellular carcinom

(*) Fully adjusted model: Energy intake, BMI, Alcohol at recruitment & lifetime alcohol intake, Physical activity index, Smoking status, Education and Diabetes and Coffee consumption

Mycotoxins for which only insignificant values have been detected are written in Italic font (Citrinin, Diacetoxyscirpenol, Fusarenon X, Sterigmatocystin).

Additional analyses identifying the most important food groups contributing to these mycotoxins’ exposures revealed relevant contributors to be cereals and cereal products (39.1%), vegetables (20.3%), and the group of fruits, nuts and seeds (11.7%). Within the other food groups, we found very low or no concentrations of any mycotoxin, except for ZEN and AFM1 that are present in dairy products and patulin present in non-alcoholic beverages (including fruit juices) (S13 Table).

The results presented in this manuscript indicates the potential importance of investigating mycotoxin exposures in Europe. The external exposure analyses using EPIC dietary questionnaire data shows possible important exposures to particular mycotoxins in European countries. Our findings indicate a potentially increased HCC risk with higher exposures to the ubiquitously present *Fusarium*-toxin DON. Interestingly, no significant associations were found with other mycotoxins including *Aspergillus*-toxins, especially AFB1, a well-established liver carcinogen designated as a Group 1 human carcinogen by IARC (due to its genotoxic and mutational effects that are considered the main mechanism of action for AFB1) and suggested to induce persistent epigenomic effects (*i*.*e*. methyl DNA-mRNA-interactions) in primary human hepatocytes associated with HCC [44]. This lack of association with AFB1 in our study may be because exposures in Europe are very low, in contrast to DON which is ubiquitous in European diets. Indeed, several studies conducted in various European countries indicate that more than 50% of cereals are contaminated with DON [45].

DON targets the ribosome and induces activation of mitogen-activated protein kinases (MAPKs), the key transducers of the ribotoxic stress response [46, 47]. Inflammation, apoptosis as well as cell cycle arrest, endoplasmic reticulum stress, oxidative stress, and autophagy of the chaperone GRP78 are the main cellular effects of DON. Pathological sequelae resulting from chronic low dose exposure include anorexia, impaired weight gain, growth hormone dysregulation, and aberrant IgA production, whereas acute high dose exposure evokes gastroenteritis, emesis, and a shock-like syndrome [48]. It has been concluded that the capacity of DON to evoke ribotoxic stress contributes significantly to its acute and chronic toxic effects *in vivo* [46]. The potential of DON to induce transcription factors in Human Hepatoma cells has been investigated [50]. DON exposure induces decrease in cell viability in a concentration-dependent manner. Treatment of the Hep-G2 liver cell line with 1 μM DON resulted in at least 75% cell viability, whereas at high-DON concentrations (10 μM) only 15% of the cells showed metabolic activity after 48 h exposure [49]. More recently an inflammatory and apoptotic effect of DON was also observed in mouse precision-cut liver slices [50].

Several *in vivo* studies also demonstrated that mice orally exposed to low a concentration of DON for 28–30 days display low-grade inflammation as measured by cytokine concentrations in the plasma and the expression of inflammatory mRNA biomarkers in different organs including liver [46, 51]. Histological analysis of DON exposed animals also indicates various types of liver damages such as portal and periportal fibrosis [40] and lymphoid depletion [52]. A study aiming to characterize the chronic effects of DON by exposing cancer-prone transgenic p53 heterozygous (*p53+/-)* male mice and p53 homozygous (*p53+/+)* male control mice reported that DON was non-carcinogenic, even in the heterozygous p53 genetic background. The hepatic and renal gene expression analyses further confirmed that chronic exposure to DON was non-inflammatory [53].

Besides DON, the cytotoxic effect on HepG2 cells of acetylated DON, namely 3-ADON and 15-ADON, was evaluated by MTT assays. It has been revealed that the strongest toxic effect was observed at 48 h expressed by an IC50 of 3.6 ±1.2μM, which was the lowest IC50 observed for 3-ADON at any exposure time [54]. The study demonstrated cytotoxic effects on the HepG2 cell line in a dose-dependent manner for 3-ADON and 15-ADON individually [54].

Moreover, a synergy has been observed between DON and other trichotechenes including the acetylated forms of DON, for both cytotoxic and inflammatory effects [55, 56]. Considering that food is co-contaminated by several trichotechenes this synergy is likely to occur in vivo in humans.

Even though DON has not been classified as to its carcinogenicity for humans, it has been shown to exacerbate the DNA damage induced by various genotoxins as measured by the expression of the marker gH2AX. This effect is observed in vitro and in vivo in combination with model genotoxins with different modes of action, including captan, a pesticide with genotoxic potential, and colibactin, a bacterial genotoxin produced by the intestinal microbiota [47, 57, 58]. It would be of interest to determine to which extend individuals of the EPIC cohort exposed to DON and developing HCC were also exposed to low levels of other genotoxic compounds.

Interestingly, our results showed a possible decreased risk for HCC risk with higher exposure to moniliformin and citrinin (both mainly found in cereals). No other epidemiological studies have reported similar inverse associations between cancer risk and higher exposure to moniliformin, an observation that requires further research. As described in the tables, only insignificant values have been detected for citrinin, as such considered as a potentially less reliable exposure assessment. Our results may be prone to residual confounding bias e.g., dietary patterns consisting of various cereal and cereal products in combination with other positive dietary aspects, which may explain the inverse association observed for moniliformin.

Important strengths of this investigation were the access to one of the largest cohort databases currently available for investigating effects of dietary exposures on cancer risk. Strengths of the EPIC study include its large sample size, its prospective design, its long follow-up, and the inclusion of participants from different European countries with harmonised data collection, especially for diet, offering a broad perspective on dietary intakes in Europe. Also, the access to the EFSA mycotoxin occurrence data derived from the European member states and the important support by the EFSA experts were strengths of this study. In addition, the sensitivity analyses performed on the different levels of these analyses underscores the quality of the results obtained in this project.

However, some limitations should be acknowledged. Limitations of this project are the dietary intake assessment methods that were used in EPIC (mostly self-reported dietary questionnaires) which may be prone to reporting bias. Indeed, diet measurement instruments are built to capture the usual dietary intakes of an individual but are still subject to imprecision and inaccuracy. For example, spices are an important source of mycotoxins but are not captured in the EPIC questionnaires which may cause imprecise estimates of the amounts of actual mycotoxins consumed. Also, the mycotoxin levels in foods notably depend on environmental factors like climate and, storage conditions, leading to important variations in mycotoxin concentrations measured in similar foods. However, in epidemiological analysis these variations can unfortunately not be considered. Furthermore, the EFSA food occurrence data has its limitations as food samples have been analysed in different laboratories available all over Europe and disposing of different laboratory infrastructures and methods available and at different timepoints; therefore, not necessarily reflecting the foods consumed in the different EPIC countries and regions. As demonstrated in Table 2, for some of the mycotoxins such as citrinin, only insignificant values had been measured/detected in the foods that were analysed by the EFSA member states, so the values in the Middle-bound scenario were purely based upon the LOD and LOQ values for these mycotoxins. Given the small number of participants exposed (above PMTDI for DON) and the small number of HCC cases, results on DON association with hepatic cancer incidence should be taken cautiously. In addition, caution is needed regarding the extrapolation of these results to the entire European population or to other populations or ethnicities worldwide since this study included volunteers that may be expected to have more health-conscious behaviours (i.e., higher intakes of fruits, vegetables and wholegrains) compared to the general population. Further, in our models, we included all the participants with available dietary intake data, but with potential missing data on other covariates replaced with a *missing* class or imputation. Although this may have induced some residual confounding bias, a complete case model would lead to a selection towards more compliant participants in an already health-conscious population. In addition, this study used a single assessment of dietary intakes at baseline. Although diet may change over time, it is usually hypothesized that this estimation reflects general eating behaviour throughout middle-aged adult life [59]. Finally, this study was based on an observational cohort. Thus, even though our models included a large range of confounding factors, residual confounding cannot be entirely ruled out.

This indirect approach that aims to assess mycotoxin exposures via food consumption data and mycotoxin occurrences in foods, also referred to as ‘external exposure’ estimation, gives the community a first insight on the global mycotoxin issue at the population level. The ‘internal exposure’ estimation takes into account additional variables such as mycotoxicokinetics and -dynamics [60]. Therefore, additional information derived from biological markers for internal exposure are needed to characterize the physiological processes involved in any potential relationship between mycotoxin exposures and cancer risk. Investigating the internal exposure is essential to understand the human mycotoxicokinetics and to exclude the possible confounding issue of heterogeneous distribution of mycotoxins in foods. Hence, a more suitable and reliable mycotoxin exposure assessment can be achieved by the direct measurement of mycotoxin exposure biomarkers. Calculating intake levels from biomarker levels is still challenging, therefore both, external as well as internal exposure assessments are recommended when investigating and tackling health effects of multiple mycotoxin exposures [61]. In addition, although some of the discussed mechanisms described for DON can be related to carcinogenesis, additional mechanistic studies are also needed for improving our understanding regarding the potential carcinogenic effects of DON exposures.

These results demonstrating potential increases in HCC risk due to chronic mycotoxin exposures can help raise awareness of these high-risk contaminants in the general public as well as product development industries and governing regulatory bodies. Several practical primary and secondary prevention strategies exist for minimizing public mycotoxin exposure, which could be highly beneficial, if the requisite political will and financial investment are applied to what remains a largely ignored global health issue [62]. It should be underlined that prevention strategies should not aim at avoiding foods that are more prone for being contaminated with mycotoxins, but rather at eliminating or reducing the contamination level of the foods by improving storage and transportation conditions and detoxification after contamination.

## Conclusions

These analyses showed greater HCC risk associated with long-term dietary exposures to DON. However, validation of these findings in other studies and via biomarkers is necessary. Further research investigating potential mechanisms underlying these putative associations is warranted. Even though DON has not been classified as to its carcinogenicity for humans, this mycotoxin presents a potential threat to human health.

## Supporting information

S1 Table Mycotoxins classified according to the IARC Monograph that identifies and evaluates environmental causes of cancer in humans. (DOCX)

S2 Table Complete list of mycotoxin groups and individual mycotoxins included in this study. (DOCX)

S3 Table Description of the external mycotoxin exposures assessed based upon dietary questionnaire data for the full EPIC cohort for lower bound values in μg/d. (DOCX)

S4 Table Description of the dietary mycotoxin exposures assessed based upon dietary questionnaire data for the full EPIC cohort for middle bound values in μg/d. (DOCX)

S5 Table Description of the external mycotoxin exposures assessed based upon dietary questionnaire data for the full EPIC cohort for lower bound values in μg/kg body weight per day. (DOCX)

S6 Table Description of the external mycotoxin exposures assessed based upon dietary questionnaire data for the full EPIC cohort for middle bound values in μg/kg body weight per day. (DOCX)

S7 Table Odds ratios (OR) and their 95% confidence intervals (CI) for the associations between mycotoxin exposures (μg/BW*day) and liver cancer risk using an adjusted model* (with and without adjustment for hepatitis). P-value of 0.01 was considered statistically significant (after Bonferroni correction).
(DOCX)

S8 Table Hazard ratios (HR) and their 95% confidence intervals (CI) for the associations between mycotoxin exposures (μg/BW*day) and liver cancer risk using an adjusted model* (without adjustment for coffee consumption). Both total HCC and the different subsites are presented (n = 450,112; HCC cases = 255 & non-cases = 449,857). P-value of 0.01 was considered statistically significant (after Bonferroni correction).
(DOCX)

S9 Table Hazard ratios (HR) and their 95% confidence intervals (CI) for the associations between mycotoxin (body weight) exposures and liver cancer risk between male and females using a fully adjusted model*. P-value of 0.01 was considered statistically significant (after Bonferroni correction).
(DOCX)

S10 Table Hazard ratios (HR) and their 95% confidence intervals (CI) for the associations between mycotoxin exposures and intrahepatic biliary tract cancer risk using a fully adjusted model*. (DOCX)

S11 Table Hazard ratios (HR) and their 95% confidence intervals (CI) for the associations between mycotoxin exposures and extra-hepatic biliary tract cancer risk using a fully adjusted model*. (DOCX)

S12 Table Hazard ratios (HR) and their 95% confidence intervals (CI) for the associations between mycotoxin exposures and gall bladder and biliary tract cancer risk using a fully adjusted model*. (DOCX)

S13 Table Sources of mycotoxins: %contributions from the main EPIC food groups. (DOCX)
