# Supplementary material for: Associations between dietary mycotoxins exposures and risk of hepatocellular carcinoma in a European cohort
Source: PLoS One. 2024 Dec 16;19(12):e0315561. doi: 10.1371/journal.pone.0315561 (PMC11649147; doi:10.1371/journal.pone.0315561)
Supplement: S1 Table — (DOCX) [file pone.0315561.s001.docx]

**S1 Table. Mycotoxins classified according to the IARC Monograph that identifies and evaluates environmental causes of cancer in humans (adapted from Claeys, L. 2022) [1].**

| **IARC classification [2]** | **Mycotoxin** | **Publication year of IARC Monograph** |
| --- | --- | --- |
| Group 1: the agent is carcinogenic to humans | AFB1, AFB2, AFG1, AFG2, AFM1 | IARC 2012 [3, 4] |
| Group 2A: the agent is probably carcinogenic to humans |  |  |
| Group 2B: the argent is possibly carcinogenic to humans | OTA  FB1, FB2  STC  Fusarin C | IARC 1993 [5]  IARC 2002 [4, 6]  IARC 1993 [5]  IARC 1976 [7] |
| Group 3: the agent is not classifiable as to its carcinogenicity to humans | DON  ZEN  Fusarenone X  CIT  PAT | IARC 1993 [5]  IARC 1986 [8] |
| Group 4: the agent is probably not carcinogenic to humans |  |  |
| Legend: aflatoxin B1 (AFB1), aflatoxin B2 (AFB2), aflatoxin G1 (AFG1), aflatoxin G2 (AFG2), aflatoxin M1 (AFM1), ochratoxin A (OTA), fumonisin B1 (FB1), fumonisin B2 (FB2), sterigmatocystin (STC), deoxynivalenol (DON), zearalenone (ZEN), citrinin (CIT), patulin (PAT) | | |

**Reference list**

1. Claeys L. Mycotoxins and human carcinogenesis : exploring causal links by exposure assessment and poly-omics designs. [Ghent, Belgium]: Ghent University. Faculty of Pharmaceutical Sciences; 2022.
2. IARC. IARC monographs on the evaluation of carcinogenic risks to humans. IARC Monogr. Eval. Carcinog. Risks to Humans. 2010. 93, 9–38.
3. IARC. Volume 100F IARC Monographs on the evaluation of carcinogenic risks to humans: chemical agents and related occupations. 2012. 100F, 9–562.
4. IARC Monographs Priorities Group. Advisory Group recommendations on priorities for the IARC Monographs. Lancet Oncol. 2019. 20, 763–764.
5. IARC. Volume 56 IARC Monographs on the evaluation of carcinogenic risk of chemicals to humans. 1993. 56.
6. IARC. Volume 82 IARC Monographs on the Evaluation of Carcinogenic Risks To Humans. 2002. 82.
7. IARC. Volume 10 IARC Monographs on the evaluation of carcinogenic risk of chemicals to man. 1976. 51, 191, 205, 245.
8. IARC. Volume 40 IARC Monographs on the evaluation of the carcinogenic risk of chemicals to humans. 1986. 40, 452.
