# Supplementary material for: Associations between dietary mycotoxins exposures and risk of hepatocellular carcinoma in a European cohort
Source: PLoS One. 2024 Dec 16;19(12):e0315561. doi: 10.1371/journal.pone.0315561 (PMC11649147; doi:10.1371/journal.pone.0315561)
Supplement: S2 Table — (DOCX) [file pone.0315561.s002.docx]

**S2 Table.** **Complete list of mycotoxin groups and individual mycotoxins included in this study.**

| **Group** | **Mycotoxin** |
| --- | --- |
| *Aflatoxins* | Aflatoxin B1 |
|  | Aflatoxin B2 |
|  | Aflatoxin G1 |
|  | Aflatoxin G2 |
|  | Aflatoxin M1 |
| *Deoxynivalenol (DON) and derivatives* | *Deoxynivalenol* |
|  | 3-Acetyl-DON |
|  | 15-Acetyl-DON |
|  | Deoxynivalenol-3-glucoside |
| *Fumonisins* | Fumonisin B1 |
|  | Fumonisin B2 |
|  | Fumonisin B3 |
| *Zearalenone and derivatives* | Zearalenone |
|  | Zen-derivatives |
|  | α-Zearalenol |
|  | β-Zearalenol |
|  | Zearalenone |
| *Alternaria toxins* | Alternariol |
|  | Alternariol mono-methylether |
|  | Altenuene |
|  | Tenuazonic acid |
|  | Altertoxin I |
|  | Tentoxin |
|  | Alternaria alternata f. sp. lycopersici toxins |
| *Enniatins* | Enniatin A |
|  | Enniatin A1 |
|  | Enniatin B |
|  | Enniatin B1 |
| *Ergot alkaloids* | Ergocornine |
|  | Ergocorninine |
|  | Ergocristine |
|  | Ergocristinine |
|  | α-Ergokryptine |
|  | α-Ergokryptinine β-Ergokryptine |
|  | Ergometrine |
|  | Ergometrinine |
|  | Ergosine |
|  | Ergosinine |
|  | Ergotamine |
|  | Ergotaminine |
| *Ochratoxins* | Ochratoxin A |
| *T2 & HT2* | HT-2 toxin |
|  | T-2 toxin |
| Individual mycotoxins | Patulin |
|  | Nivalenol |
|  | Diacetoxyscirpenol |
|  | Fusarenon-X |
|  | Moniliformin |
|  | Citrinin |
|  | Beauvericin |
|  | Sterigmatocystins |
