# Supplementary material for: Associations between dietary mycotoxins exposures and risk of hepatocellular carcinoma in a European cohort
Source: PLoS One. 2024 Dec 16;19(12):e0315561. doi: 10.1371/journal.pone.0315561 (PMC11649147; doi:10.1371/journal.pone.0315561)
Supplement: S3 Table — (DOCX) [file pone.0315561.s003.docx]

***S3 Table.*** **Description of the dietary mycotoxin exposures assessed based upon dietary questionnaire data for the full EPIC cohort for lower bound values in µg/d.**

|  |  |  | **Lower Bound (LB) - µg/d** | | | | |
| --- | --- | --- | --- | --- | --- | --- | --- |
|  |  |  |  |  |  |  |  |
| **Non-case (0)/Case (1)** | **Label** | **N** | **Mean** | **Std** | **Median** | **25th** | **75th** |
|  |  |  |  | **Dev** |  | **Pctl** | **Pctl** |
| 0 | Beauvericin | 449857 | 0 | 0 | 0 | 0 | 0 |
| 1 | Beauvericin | 255 | 0 | 0 | 0 | 0 | 0 |
| *0* | *Citrinin* | *449857* | *0* | *0* | *0* | *0* | *0* |
| *1* | *Citrinin* | *255* | *0* | *0* | *0* | *0* | *0* |
| *0* | *Diacetoxyscirpenol* | *449857* | *0* | *0* | *0* | *0* | *0* |
| *1* | *Diacetoxyscirpenol* | *255* | *0* | *0* | *0* | *0* | *0* |
| *0* | *Fusarenon X* | *449857* | *0* | *0* | *0* | *0* | *0* |
| *1* | *Fusarenon X* | *255* | *0* | *0* | *0* | *0* | *0* |
| 0 | Moniliformine | 449857 | 0.15 | 0.62 | 0 | 0 | 0 |
| 1 | Moniliformine | 255 | 0.15 | 0.55 | 0 | 0 | 0.01 |
| 0 | Nivalenol | 449857 | 0 | 0.01 | 0 | 0 | 0 |
| 1 | Nivalenol | 255 | 0 | 0 | 0 | 0 | 0 |
| 0 | Patulin | 449857 | 0.18 | 0.56 | 0 | 0 | 0.11 |
| 1 | Patulin | 255 | 0.33 | 0.96 | 0 | 0 | 0.13 |
| *0* | *Sterigmatocystins* | *449857* | *0* | *0* | *0* | *0* | *0* |
| *1* | *Sterigmatocystins* | *255* | *0* | *0* | *0* | *0* | *0* |
| 0 | Aflatoxins | 449857 | 0.01 | 0.02 | 0.01 | 0 | 0.01 |
| 1 | Aflatoxins | 255 | 0.01 | 0.02 | 0 | 0 | 0.02 |
| 0 | Alternaria toxins | 449857 | 0.49 | 0.74 | 0.24 | 0.08 | 0.64 |
| 1 | Alternaria toxins | 255 | 0.84 | 1.44 | 0.34 | 0.11 | 0.85 |
| 0 | Deoxynivalenol and derivatives | 449857 | 5.11 | 4.69 | 3.86 | 1.71 | 7.07 |
| 1 | Deoxynivalenol and derivatives | 255 | 6.02 | 6.5 | 4.25 | 1.86 | 8.29 |
| 0 | Ergot alkaloids | 449857 | 1.09 | 2.37 | 0.36 | 0.08 | 1.09 |
| 1 | Ergot alkaloids | 255 | 1.28 | 2.89 | 0.37 | 0.06 | 1 |
| 0 | Enniatins | 449857 | 3.19 | 3.43 | 1.99 | 0.94 | 4.23 |
| 1 | Enniatins | 255 | 3.9 | 3.84 | 2.66 | 1.22 | 5.15 |
| 0 | Fumonisins | 449857 | 3.29 | 3.91 | 2.34 | 1.14 | 4.29 |
| 1 | Fumonisins | 255 | 3.27 | 4.94 | 1.99 | 1.02 | 3.77 |
| 0 | Fusarium Toxins | 449857 | 9.29 | 6.49 | 7.75 | 5.03 | 11.77 |
| 1 | Fusarium Toxins | 255 | 10.24 | 9.11 | 7.95 | 4.82 | 12.67 |
| 0 | Ochratoxins | 449857 | 0.06 | 0.07 | 0.04 | 0.02 | 0.08 |
| 1 | Ochratoxins | 255 | 0.06 | 0.09 | 0.03 | 0.01 | 0.07 |
| 0 | T-2/HT-2 toxins | 449857 | 0.06 | 0.23 | 0 | 0 | 0.01 |
| 1 | T-2/HT-2 toxins | 255 | 0.05 | 0.16 | 0 | 0 | 0.01 |
| 0 | Zearalenone & derivatives | 449857 | 0.83 | 1.45 | 0.42 | 0.07 | 0.91 |
| 1 | Zearalenone & derivatives | 255 | 0.9 | 1.6 | 0.44 | 0.1 | 1.03 |
| 0 | Mycotoxins | 449857 | 14.46 | 9.57 | 12.19 | 7.91 | 18.46 |
| 1 | Mycotoxins | 255 | 16.81 | 12.13 | 13.82 | 8.92 | 22.02 |

Mycotoxins for which only insignificant values have been detected are written in Italic font (Citrinin, Diacetoxyscirpenol, Fusarenon X, Sterigmatocystin).

No missing values
