# Supplementary material for: Associations between dietary mycotoxins exposures and risk of hepatocellular carcinoma in a European cohort
Source: PLoS One. 2024 Dec 16;19(12):e0315561. doi: 10.1371/journal.pone.0315561 (PMC11649147; doi:10.1371/journal.pone.0315561)
Supplement: S4 Table — (DOCX) [file pone.0315561.s004.docx]

***S4 Table.*** **Description of the dietary mycotoxin exposures assessed based upon dietary questionnaire data for the full EPIC cohort for middle bound values in µg/d.**

|  |  |  | **Middle Bound (MB) - µg/d** | | | | |
| --- | --- | --- | --- | --- | --- | --- | --- |
|  |  |  |  |  |  |  |  |
| **Non-case (0)/**  **Case (1)** | **Label** | **N** | **Mean** | **Std** | **Median** | **25th** | **75th** |
|  |  |  |  | **Dev** |  | **Pctl** | **Pctl** |
| 0 | Beauvericin | 449857 | 0 | 0.01 | 0 | 0 | 0 |
| 1 | Beauvericin | 255 | 0.01 | 0.01 | 0 | 0 | 0.01 |
| *0* | *Citrinin* | *449857* | *0* | *0* | *0* | *0* | *0* |
| *1* | *Citrinin* | *255* | *0* | *0* | *0* | *0* | *0* |
| *0* | *Diacetoxyscirpenol* | *449857* | *2.25* | *1.15* | *2.05* | *1.54* | *2.7* |
| *1* | *Diacetoxyscirpenol* | *255* | *2.54* | *2.54* | *2.03* | *1.46* | *2.92* |
| *0* | *Fusarenon X* | *449857* | *1.1* | *0.78* | *0.91* | *0.53* | *1.49* |
| *1* | *Fusarenon X* | *255* | *1.42* | *1* | *1.18* | *0.69* | *1.95* |
| 0 | Moniliformine | 449857 | 0.24 | 0.62 | 0.08 | 0.03 | 0.18 |
| 1 | Moniliformine | 255 | 0.24 | 0.56 | 0.08 | 0.02 | 0.17 |
| 0 | Nivalenol | 449857 | 2.11 | 1.31 | 1.84 | 1.18 | 2.73 |
| 1 | Nivalenol | 255 | 2.43 | 1.61 | 2.13 | 1.28 | 3.08 |
| 0 | Patulin | 449857 | 1.01 | 0.85 | 0.79 | 0.5 | 1.24 |
| 1 | Patulin | 255 | 1.46 | 1.49 | 0.9 | 0.58 | 1.86 |
| *0* | *Sterigmatocystins* | *449857* | *0.09* | *0.09* | *0.06* | *0.04* | *0.1* |
| *1* | *Sterigmatocystins* | *255* | *0.07* | *0.05* | *0.05* | *0.03* | *0.09* |
| 0 | Aflatoxins | 449857 | 0.22 | 0.1 | 0.2 | 0.15 | 0.27 |
| 1 | Aflatoxins | 255 | 0.21 | 0.11 | 0.19 | 0.13 | 0.25 |
| 0 | Alternaria toxins | 449857 | 13.24 | 7.07 | 12.25 | 8.15 | 17.12 |
| 1 | Alternaria toxins | 255 | 16.33 | 7.74 | 15.18 | 10.56 | 20.62 |
| 0 | Deoxynivalenol and derivatives | 449857 | 16.34 | 8.53 | 14.76 | 10.45 | 20.34 |
| 1 | Deoxynivalenol and derivatives | 255 | 20.25 | 14.78 | 17.82 | 12.45 | 24.24 |
| 0 | Ergot alkaloids | 449857 | 4.83 | 4.55 | 3.61 | 1.74 | 6.43 |
| 1 | Ergot alkaloids | 255 | 5.76 | 5.11 | 4.57 | 2.7 | 7.19 |
| 0 | Enniatins | 449857 | 3.25 | 3.47 | 2.03 | 0.96 | 4.33 |
| 1 | Enniatins | 255 | 3.96 | 3.86 | 2.75 | 1.23 | 5.27 |
| 0 | Fumonisins | 449857 | 16.28 | 8.47 | 14.62 | 10.51 | 20.14 |
| 1 | Fumonisins | 255 | 18.7 | 12.02 | 15.79 | 10.73 | 23.16 |
| 0 | Fusarium Toxins | 449857 | 40.7 | 18.38 | 37.6 | 27.91 | 49.89 |
| 1 | Fusarium Toxins | 255 | 48.29 | 30.23 | 43 | 29.68 | 58.53 |
| 0 | Ochratoxins | 449857 | 0.15 | 0.08 | 0.13 | 0.1 | 0.18 |
| 1 | Ochratoxins | 255 | 0.16 | 0.1 | 0.13 | 0.1 | 0.18 |
| 0 | T-2/HT-2 toxins | 449857 | 1.26 | 0.75 | 1.13 | 0.74 | 1.62 |
| 1 | T-2/HT-2 toxins | 255 | 1.57 | 0.99 | 1.34 | 0.86 | 2.08 |
| 0 | Zearalenone & derivatives | 449857 | 2.46 | 1.7 | 2.04 | 1.5 | 2.84 |
| 1 | Zearalenone & derivatives | 255 | 2.79 | 2.56 | 2.14 | 1.51 | 3.23 |
| 0 | Mycotoxins | 449857 | 63.73 | 28.85 | 58.93 | 43.32 | 78.66 |
| 1 | Mycotoxins | 255 | 76.47 | 39.63 | 70.13 | 49.34 | 91.65 |

Mycotoxins for which only insignificant values have been detected are written in Italic font (Citrinin, Diacetoxyscirpenol, Fusarenon X, Sterigmatocystin).

No missing values
