# Supplementary material for: Associations between dietary mycotoxins exposures and risk of hepatocellular carcinoma in a European cohort
Source: PLoS One. 2024 Dec 16;19(12):e0315561. doi: 10.1371/journal.pone.0315561 (PMC11649147; doi:10.1371/journal.pone.0315561)
Supplement: S6 Table — (DOCX) [file pone.0315561.s006.docx]

**S6 Table. Description of the external mycotoxin exposures assessed based upon dietary questionnaire data for the full EPIC cohort for middle bound values in µg/kg body weight per day.**

|  |  |  | **Middle Bound (MB) - µg/kg body weight*d** | | | | |
| --- | --- | --- | --- | --- | --- | --- | --- |
|  |  |  |  |  |  |  |  |
| **Non-case (0)/**  **Case (1)** | **Label** | **N** | **Mean** | **Std** | **Median** | **25th** | **75th** |
|  |  |  |  | **Dev** |  | **Pctl** | **Pctl** |
| 0 | Beauvericin | 449857 | 0 | 0 | 0 | 0 | 0 |
| 1 | Beauvericin | 255 | 0 | 0 | 0 | 0 | 0 |
| *0* | *Citrinin* | *449857* | *0* | *0* | *0* | *0* | *0* |
| *1* | *Citrinin* | *255* | *0* | *0* | *0* | *0* | *0* |
| *0* | *Diacetoxyscirpenol* | *449857* | *0.03* | *0.02* | *0.03* | *0.02* | *0.04* |
| *1* | *Diacetoxyscirpenol* | *255* | *0.03* | *0.03* | *0.03* | *0.02* | *0.04* |
| *0* | *Fusarenon X* | *449857* | *0.02* | *0.01* | *0.01* | *0.01* | *0.02* |
| *1* | *Fusarenon X* | *255* | *0.02* | *0.01* | *0.02* | *0.01* | *0.02* |
| 0 | Moniliformine | 449857 | 0 | 0.01 | 0 | 0 | 0 |
| 1 | Moniliformine | 255 | 0 | 0.01 | 0 | 0 | 0 |
| 0 | Nivalenol | 449857 | 0.03 | 0.02 | 0.03 | 0.02 | 0.04 |
| 1 | Nivalenol | 255 | 0.03 | 0.02 | 0.03 | 0.02 | 0.04 |
| 0 | Patulin | 449857 | 0.01 | 0.01 | 0.01 | 0.01 | 0.02 |
| 1 | Patulin | 255 | 0.02 | 0.02 | 0.01 | 0.01 | 0.02 |
| *0* | *Sterigmatocystins* | *449857* | *0* | *0* | *0* | *0* | *0* |
| *1* | *Sterigmatocystins* | *255* | *0* | *0* | *0* | *0* | *0* |
| 0 | Aflatoxins | 449857 | 0 | 0 | 0 | 0 | 0 |
| 1 | Aflatoxins | 255 | 0 | 0 | 0 | 0 | 0 |
| 0 | Alternaria toxins | 449857 | 0.19 | 0.1 | 0.18 | 0.12 | 0.25 |
| 1 | Alternaria toxins | 255 | 0.21 | 0.1 | 0.2 | 0.14 | 0.26 |
| 0 | Deoxynivalenol and derivatives | 449857 | 0.24 | 0.13 | 0.22 | 0.15 | 0.3 |
| 1 | Deoxynivalenol and derivatives | 255 | 0.26 | 0.17 | 0.23 | 0.16 | 0.3 |
| 0 | Ergot alkaloids | 449857 | 0.07 | 0.07 | 0.05 | 0.03 | 0.09 |
| 1 | Ergot alkaloids | 255 | 0.07 | 0.06 | 0.06 | 0.03 | 0.1 |
| 0 | Enniatins | 449857 | 0.05 | 0.05 | 0.03 | 0.01 | 0.06 |
| 1 | Enniatins | 255 | 0.05 | 0.05 | 0.03 | 0.01 | 0.07 |
| 0 | Fumonisins | 449857 | 0.24 | 0.13 | 0.21 | 0.15 | 0.3 |
| 1 | Fumonisins | 255 | 0.24 | 0.14 | 0.2 | 0.15 | 0.29 |
| 0 | Fusarium Toxins | 449857 | 0.6 | 0.28 | 0.55 | 0.4 | 0.74 |
| 1 | Fusarium Toxins | 255 | 0.61 | 0.34 | 0.55 | 0.39 | 0.75 |
| 0 | Ochratoxins | 449857 | 0 | 0 | 0 | 0 | 0 |
| 1 | Ochratoxins | 255 | 0 | 0 | 0 | 0 | 0 |
| 0 | T-2/HT-2 toxins | 449857 | 0.02 | 0.01 | 0.02 | 0.01 | 0.02 |
| 1 | T-2/HT-2 toxins | 255 | 0.02 | 0.01 | 0.02 | 0.01 | 0.03 |
| 0 | Zearalenone & derivatives | 449857 | 0.04 | 0.02 | 0.03 | 0.02 | 0.04 |
| 1 | Zearalenone & derivatives | 255 | 0.04 | 0.03 | 0.03 | 0.02 | 0.04 |
| 0 | Mycotoxins | 449857 | 0.93 | 0.43 | 0.86 | 0.62 | 1.16 |
| 1 | Mycotoxins | 255 | 0.97 | 0.46 | 0.9 | 0.67 | 1.23 |

Mycotoxins for which only insignificant values have been detected are written in Italic font (Citrinin, Diacetoxyscirpenol, Fusarenon X, Sterigmatocystin).

No missing values
