# Supplementary material for: Associations between dietary mycotoxins exposures and risk of hepatocellular carcinoma in a European cohort
Source: PLoS One. 2024 Dec 16;19(12):e0315561. doi: 10.1371/journal.pone.0315561 (PMC11649147; doi:10.1371/journal.pone.0315561)
Supplement: S7 Table — P-value of 0.01 was considered statistically significant (after Bonferroni correction). (DOCX) [file pone.0315561.s007.docx]

**S7 Table. Odds ratios (OR) and their 95 % confidence intervals (CI) for the associations between mycotoxin exposures (μg/BW*day) and liver cancer risk using an adjusted model* (with and without adjustment for hepatitis).** P-value of 0.01 was considered statistically significant (after Bonferroni correction).

| Mycotoxin | Tertiles |  | HCC with hepatitis |  | HCC without hepatitis | |  |
| --- | --- | --- | --- | --- | --- | --- | --- |
|  |  | **N** | **OR (95%CI)** | **Trend test** | **N** | **OR (95%CI)** | **Trend test** |
| Ergot alkaloids | T1 | 86/157 | ref. | . | 86/157 | ref. | . |
|  | T2 | 74/157 | 1.21 (0.63-2.33) | . | 74/157 | 1.14 (0.62-2.10) | . |
|  | T3 | 76/156 | 1.91 (0.87-4.22) | 0.11 | 76/156 | 1.48 (0.73-3.02) | 0.278 |
| Ochratoxins | T1 | 98/157 | ref. | . | 98/157 | ref. | . |
|  | T2 | 61/157 | 1.15 (0.65-2.02) | . | 61/157 | 0.94 (0.55-1.60) | . |
|  | T3 | 77/156 | 2.04 (1.05-3.98) | 0.043 | 77/156 | 1.47 (0.80-2.70) | 0.242 |
| Aflatoxins | T1 | 99/157 | ref. | . | 99/157 | ref. | . |
|  | T2 | 69/157 | 0.77 (0.43-1.39) | . | 69/157 | 0.65 (0.37-1.14) | . |
|  | T3 | 68/156 | 1.27 (0.66-2.43) | 0.488 | 68/156 | 1.09 (0.60-2.00) | 0.809 |
| Patulin | T1 | 85/157 | ref. | . | 85/157 | ref. | . |
|  | T2 | 59/157 | 0.83 (0.46-1.50) | . | 59/157 | 0.83 (0.48-1.43) | . |
|  | T3 | 92/156 | 1.21 (0.67-2.19) | 0.51 | 92/156 | 1.16 (0.66-2.02) | 0.598 |
| Deoxynivalenol and derivatives | T1 | 80/157 | ref. | . | 80/157 | ref. | . |
|  | T2 | 65/157 | 1.28 (0.68-2.39) | . | 65/157 | 1.30 (0.73-2.32) | . |
|  | T3 | 91/156 | 2.25 (1.11-4.57) | 0.024 | 91/156 | 1.98 (1.04-3.76) | 0.037 |
| T-2/HT-2 toxins | T1 | 89/157 | ref. | . | 89/157 | ref. | . |
|  | T2 | 58/157 | 0.76 (0.43-1.35) | . | 58/157 | 0.80 (0.47-1.37) | . |
|  | T3 | 89/156 | 1.35 (0.69-2.64) | 0.433 | 89/156 | 1.52 (0.82-2.80) | 0.203 |
| Nivalenol | T1 | 84/157 | ref. | . | 84/157 | ref. | . |
|  | T2 | 61/157 | 0.72 (0.38-1.39) | . | 61/157 | 0.84 (0.45-1.57) | . |
|  | T3 | 91/156 | 1.38 (0.68-2.79) | 0.326 | 91/156 | 1.37 (0.71-2.63) | 0.286 |
| Fumonisins | T1 | 91/157 | ref. | . | 91/157 | ref. | . |
|  | T2 | 65/157 | 0.77 (0.42-1.42) | . | 65/157 | 0.80 (0.46-1.40) | . |
|  | T3 | 80/156 | 1.46 (0.76-2.79) | 0.246 | 80/156 | 1.36 (0.73-2.50) | 0.323 |
| Diacetoxyscirpenol | T1 | 100/157 | ref. | . | 100/157 | ref. | . |
|  | T2 | 69/157 | 0.86 (0.49-1.53) | . | 69/157 | 1.03 (0.61-1.75) | . |
|  | T3 | 67/156 | 1.16 (0.57-2.36) | 0.755 | 67/156 | 1.20 (0.62-2.31) | 0.607 |
| Zearalenone & derivatives | T1 | 92/157 | ref. | . | 92/157 | ref. | . |
|  | T2 | 80/157 | 0.80 (0.45-1.45) | . | 80/157 | 0.85 (0.49-1.49) | . |
|  | T3 | 64/156 | 0.49 (0.23-1.06) | 0.075 | 64/156 | 0.52 (0.25-1.07) | 0.085 |
| Fusarium Toxins | T1 | 84/157 | ref. | . | 84/157 | ref. | . |
|  | T2 | 69/157 | 1.14 (0.64-2.03) | . | 69/157 | 1.23 (0.71-2.12) | . |
|  | T3 | 83/156 | 1.67 (0.83-3.33) | 0.153 | 83/156 | 1.76 (0.93-3.33) | 0.083 |
| Fusarenon X | T1 | 81/157 | ref. | . | 81/157 | ref. | . |
|  | T2 | 69/157 | 1.07 (0.61-1.89) | . | 69/157 | 0.99 (0.58-1.70) | . |
|  | T3 | 86/156 | 1.84 (0.92-3.69) | 0.097 | 86/156 | 1.61 (0.84-3.08) | 0.168 |
| Sterigmatocystins | T1 | 95/157 | ref. | . | 95/157 | ref. | . |
|  | T2 | 84/157 | 1.07 (0.63-1.84) | . | 84/157 | 1.21 (0.73-2.01) | . |
|  | T3 | 57/156 | 0.55 (0.27-1.11) | 0.155 | 57/156 | 0.56 (0.29-1.07) | 0.147 |
| Moniliformine | T1 | 102/157 | ref. | . | 102/157 | ref. | . |
|  | T2 | 75/157 | 0.83 (0.48-1.43) | . | 75/157 | 0.85 (0.51-1.42) | . |
|  | T3 | 59/156 | 0.57 (0.28-1.14) | 0.116 | 59/156 | 0.51 (0.26-0.97) | 0.051 |
| Alternaria toxins | T1 | 82/157 | ref. | . | 82/157 | ref. | . |
|  | T2 | 79/157 | 1.36 (0.73-2.52) | . | 79/157 | 1.40 (0.79-2.49) | . |
|  | T3 | 75/156 | 2.06 (0.88-4.83) | 0.098 | 75/156 | 1.83 (0.84-3.97) | 0.123 |
| Citrinin | T1 | 99/157 | ref. | . | 99/157 | ref. | . |
|  | T2 | 80/157 | 0.88 (0.51-1.51) | . | 80/157 | 0.87 (0.52-1.44) | . |
|  | T3 | 57/156 | 0.55 (0.29-1.02) | 0.067 | 57/156 | 0.54 (0.30-0.96) | 0.042 |
| Enniatins | T1 | 90/157 | ref. | . | 90/157 | ref. | . |
|  | T2 | 66/157 | 1.00 (0.57-1.79) | . | 66/157 | 0.87 (0.50-1.50) | . |
|  | T3 | 80/156 | 1.03 (0.44-2.41) | 0.957 | 80/156 | 0.96 (0.45-2.06) | 0.849 |
| Sum of Mycotoxins | T1 | 80/157 | ref. | . | 80/157 | ref. | . |
|  | T2 | 73/157 | 1.33 (0.72-2.43) | . | 73/157 | 1.33 (0.75-2.38) | . |
|  | T3 | 83/156 | 1.88 (0.87-4.05) | 0.108 | 83/156 | 1.96 (0.98-3.93) | 0.058 |
| Sum of Mycotoxins using z-scores | T1 | 86/157 | ref. | . | 86/157 | ref. | . |
|  | T2 | 74/157 | 1.52 (0.84-2.75) | . | 74/157 | 1.37 (0.79-2.39) | . |
|  | T3 | 76/156 | 1.95 (0.89-4.28) | 0.086 | 76/156 | 1.91 (0.93-3.91) | 0.076 |

T1; Tertile 1, T2; Tertile 2, T3; Tertile 3, HCC; Hepatocellular carcinom

(*) Adjusted model: Energy intake, BMI, Alcohol at recruitment & lifetime alcohol intake, Physical activity index, Smoking status, Education and Diabetes

Mycotoxins for which only insignificant values have been detected are written in Italic font (Citrinin, Diacetoxyscirpenol, Fusarenon X, Sterigmatocystins).
