# Supplementary material for: Associations between dietary mycotoxins exposures and risk of hepatocellular carcinoma in a European cohort
Source: PLoS One. 2024 Dec 16;19(12):e0315561. doi: 10.1371/journal.pone.0315561 (PMC11649147; doi:10.1371/journal.pone.0315561)
Supplement: S8 Table — Both total HCC and the different subsites are presented (n = 450,112; HCC cases = 255 & non-cases = 449,857). P-value of 0.01 was considered statistically significant (after Bonferroni correction). (DOCX) [file pone.0315561.s008.docx]

**S8 Table.** **Hazard ratios (HR) and their 95 % confidence intervals (CI) for the associations between mycotoxin exposures (μg/BW*day) and liver cancer risk using an adjusted model* (without adjustment for coffee consumption).** Both total HCC and the different subsites are presented (n=450,112; HCC cases=255 & non-cases=449,857). P-value of 0.01 was considered statistically significant (after Bonferroni correction).

| **Mycotoxin** | **MB (middle bound)** | **Cases** | **HR** | **Probability Chi Square test** | | **Trend Test** |
| --- | --- | --- | --- | --- | --- | --- |
|  | **Tertiles** |  |  |  |  |  |
| Ergot alkaloids | . | 255 | 1.02 (0.86-1.22) | | 0.8006 | . |
|  | 1 | 72 | 1 (Ref.) | | . | . |
|  | 2 | 96 | 0.98 (0.63-1.54) | | 0.9340 | 0.7589 |
|  | 3 | 87 | 1.07 (0.63-1.82) | | 0.7940 | . |
| Ochratoxins | . | 255 | 0.97 (0.78-1.21) | | 0.7903 | . |
|  | 1 | 105 | 1 (Ref.) | | . | . |
|  | 2 | 79 | 0.81 (0.56-1.16) | | 0.2423 | 0.3295 |
|  | 3 | 71 | 0.81 (0.53-1.25) | | 0.3461 | . |
| Aflatoxins | . | 255 | 0.96 (0.75-1.21) | | 0.7172 | . |
|  | 1 | 114 | 1 (Ref.) | | . | . |
|  | 2 | 85 | 0.90 (0.63-1.29) | | 0.5601 | 0.4901 |
|  | 3 | 56 | 0.86 (0.54-1.36) | | 0.5071 | . |
| Patulin | . | 255 | 1.29 (1.17-1.43) | | <.0001 | . |
|  | 1 | 82 | 1 (Ref.) | | . | . |
|  | 2 | 78 | 0.93 (0.64-1.35) | | 0.6946 | 0.1047 |
|  | 3 | 95 | 1.35 (0.93-1.96) | | 0.1134 | . |
| Deoxynivalenol and derivatives | . | 255 | 1.12 (0.96-1.30) | | 0.1411 | . |
|  | 1 | 85 | 1 (Ref.) | | . | . |
|  | 2 | 74 | 1.24 (0.83-1.85) | | 0.3002 | **0.0053** |
|  | 3 | 96 | 1.95 (1.22-3.14) | | **0.0057** | . |
| T-2/HT-2 toxins | . | 255 | 1.20 (1.03-1.40) | | 0.0200 | . |
|  | 1 | 84 | 1 (Ref.) | | . | . |
|  | 2 | 68 | 0.85 (0.57-1.26) | | 0.4146 | 0.1492 |
|  | 3 | 103 | 1.31 (0.88-1.95) | | 0.1866 | . |
| Nivalenol | . | 255 | 1.05 (0.87-1.27) | | 0.5829 | . |
|  | 1 | 88 | 1 (Ref.) | | . | . |
|  | 2 | 77 | 0.89 (0.60-1.33) | | 0.5702 | 0.1705 |
|  | 3 | 90 | 1.37 (0.87-2.16) | | 0.1775 | . |
| Fumonisins | . | 255 | 1.08 (0.90-1.30) | | 0.3834 | . |
|  | 1 | 97 | 1 (Ref.) | | . | . |
|  | 2 | 74 | 0.98 (0.67-1.45) | | 0.9362 | 0.3952 |
|  | 3 | 84 | 1.21 (0.78-1.89) | | 0.3991 | . |
| *Diacetoxyscirpenol* | *.* | *255* | *0.98 (0.86-1.12)* | | *0.7842* | *.* |
|  | *1* | *109* | *1 (Ref.)* | | *.* | *.* |
|  | *2* | *76* | *0.98 (0.67-1.43)* | | *0.9155* | *0.9856* |
|  | *3* | *70* | *1.01 (0.63-1.61)* | | *0.9754* | *.* |
| Zearalenone & derivatives | . | 255 | 1.00 (0.82-1.22) | | 0.9929 | . |
|  | 1 | 104 | 1 (Ref.) | | . | . |
|  | 2 | 81 | 0.92 (0.63-1.34) | | 0.6574 | 0.2581 |
|  | 3 | 70 | 0.76 (0.47-1.22) | | 0.2532 | . |
| Fusarium Toxins | . | 255 | 1.12 (0.94-1.33) | | 0.2100 | . |
|  | 1 | 89 | 1 (Ref.) | | . | . |
|  | 2 | 80 | 1.24 (0.84-1.83) | | 0.2852 | 0.0855 |
|  | 3 | 86 | 1.52 (0.94-2.43) | | 0.0856 | . |
| *Fusarenon X* | *.* | *255* | *1.19 (0.98-1.44)* | | *0.0760* | *.* |
|  | *1* | *80* | *1 (Ref.)* | | *.* | *.* |
|  | *2* | *79* | *1.09 (0.74-1.60)* | | *0.6746* | *0.0880* |
|  | *3* | *96* | *1.48 (0.94-2.33)* | | *0.0892* | *.* |
| *Sterigmatocystins* | *.* | *255* | *0.79 (0.55-1.13)* | | *0.1906* | *.* |
|  | *1* | *111* | *1 (Ref.)* | | *.* | *.* |
|  | *2* | *84* | *1.01 (0.73-1.41)* | | *0.9298* | *0.1002* |
|  | *3* | *60* | *0.65 (0.42-1.02)* | | *0.0584* | *.* |
| Moniliformine | . | 255 | 0.98 (0.80-1.21) | | 0.8652 | . |
|  | 1 | 102 | 1 (Ref.) | | . | . |
|  | 2 | 81 | 0.82 (0.58-1.16) | | 0.2639 | 0.0377 |
|  | 3 | 72 | 0.65 (0.43-0.98) | | 0.0396 | . |
| Alternaria toxins | . | 255 | 1.18 (0.95-1.47) | | 0.1393 | . |
|  | 1 | 69 | 1 (Ref.) | | . | . |
|  | 2 | 98 | 1.55 (1.04-2.32) | | 0.0316 | 0.0675 |
|  | 3 | 88 | 1.62 (0.98-2.68) | | 0.0613 | . |
| *Citrinin* | *.* | *255* | *0.82 (0.00-2E162)* | | *0.0637* | *.* |
|  | *1* | *99* | *1 (Ref.)* | | *.* | *.* |
|  | *2* | *86* | *0.89 (0.63-1.26)* | | *0.5101* | ***0.0115*** |
|  | *3* | *70* | *0.58 (0.38-0.88)* | | ***0.0101*** | *.* |
| Enniatins | . | 255 | 0.99 (0.78-1.26) | | 0.9633 | . |
|  | 1 | 83 | 1 (Ref.) | | . | . |
|  | 2 | 80 | 0.99 (0.67-1.47) | | 0.9760 | 0.7952 |
|  | 3 | 92 | 1.09 (0.63-1.86) | | 0.7670 | . |
| Sum of Mycotoxins | . | 255 | 1.17 (0.96-1.43) | | 0.1276 | . |
|  | 1 | 78 | 1 (Ref.) | | . | . |
|  | 2 | 87 | 1.44 (0.96-2.15) | | 0.0781 | **0.0360** |
|  | 3 | 90 | 1.72 (1.04-2.84) | | **0.0350** | . |
| Sum of Mycotoxins using z-scores | . | 255 | 1.05 (0.85-1.31) | | 0.6509 | . |
|  | 1 | 86 | 1 (Ref.) | | . | . |
|  | 2 | 86 | 1.10 (0.74-1.63) | | 0.6313 | 0.5565 |
|  | 3 | 83 | 1.16 (0.71-1.91) | | 0.5578 | . |

(*) Adjusted model: Energy intake, BMI, Alcohol at recruitment & lifetime alcohol intake, Physical activity index, Smoking status, Education and Diabetes

Mycotoxins for which only insignificant values have been detected are written in Italic font (Citrinin, Diacetoxyscirpenol, Fusarenon X, Sterigmatocystins).
