# Supplementary material for: Associations between dietary mycotoxins exposures and risk of hepatocellular carcinoma in a European cohort
Source: PLoS One. 2024 Dec 16;19(12):e0315561. doi: 10.1371/journal.pone.0315561 (PMC11649147; doi:10.1371/journal.pone.0315561)
Supplement: S9 Table — P-value of 0.01 was considered statistically significant (after Bonferroni correction). (DOCX) [file pone.0315561.s009.docx]

**S9 Table.** ***Hazard ratios (HR) and their 95 % confidence intervals (CI) for the associations between mycotoxin (body weight) exposures and liver cancer risk between male and females using a fully adjusted model*.*** P-value of 0.01 was considered statistically significant (after Bonferroni correction).

|  | **MB (middle bound)** | **HCC (body weight)** | | | | | | | | | |
| --- | --- | --- | --- | --- | --- | --- | --- | --- | --- | --- | --- |
| **Mycotoxins** | **Mycotoxins μg/BW/day** | **Male Cases**  **162** | HR | **95%CI** | **Probability Chi Square** | **Trend test** | **Female Cases 93** | **HR** | **95%CI** | **Probability Chi Square** | **Trend test** |
| Ergot alkaloids | Per 1 SD increase | 162 | 1.06 | 0.85-1.32 | 0.6172 | . | 93 | 0.98 | 0.73-1.32 | 0.9009 | . |
|  | T1 | 45 | 1 | Ref. | . | . | 27 | 1 | Ref. | . | . |
|  | T2 | 70 | 1.21 | 0.68-2.14 | 0.5249 | 0.4927 | 26 | 0.68 | 0.32-1.45 | 0.3184 | 0.8807 |
|  | T3 | 47 | 1.29 | 0.64-2.63 | 0.4767 | . | 40 | 0.89 | 0.39-2.00 | 0.7756 | . |
| Ochratoxins | Per 1 SD increase | 162 | 0.99 | 0.74-1.32 | 0.9497 | . | 93 | 1.06 | 0.83-1.35 | 0.6429 | . |
|  | T1 | 76 | 1 | Ref. | . | . | 29 | 1 | Ref. | . | . |
|  | T2 | 43 | 0.68 | 0.43-1.09 | 0.1067 | 0.5967 | 36 | 1.5 | 0.82-2.76 | 0.1905 | 0.6874 |
|  | T3 | 43 | 0.88 | 0.51-1.52 | 0.6541 | . | 28 | 1.15 | 0.53-2.47 | 0.7208 | . |
| Aflatoxins | Per 1 SD increase | 162 | 1.15 | 0.86-1.55 | 0.3408 | . | 93 | 0.74 | 0.50-1.09 | 0.1220 | . |
|  | T1 | 76 | 1 | Ref. | . | . | 38 | 1 | Ref. | . | . |
|  | T2 | 50 | 0.91 | 0.58-1.43 | 0.6794 | 0.9931 | 35 | 0.99 | 0.55-1.81 | 0.9836 | 0.3957 |
|  | T3 | 36 | 1.01 | 0.58-1.77 | 0.9638 | . | 20 | 0.68 | 0.30-1.54 | 0.3532 | . |
| Patulin | Per 1 SD increase | 162 | 1.33 | 1.18-1.48 | <.0001 | . | 93 | 1.1 | 0.85-1.42 | 0.4774 | . |
|  | T1 | 48 | 1 | Ref. | . | . | 34 | 1 | Ref. | . | . |
|  | T2 | 44 | 1.02 | 0.62-1.68 | 0.9243 | 0.0301 | 34 | 0.75 | 0.42-1.35 | 0.3395 | 0.4170 |
|  | T3 | 70 | 1.65 | 1.03-2.65 | 0.0368 | . | 25 | 0.78 | 0.41-1.47 | 0.4384 | . |
| Deoxynivalenol and derivatives | Per 1 SD increase | 162 | 1.11 | 0.93-1.32 | 0.2377 | . | 93 | 1.15 | 0.76-1.74 | 0.5051 | . |
|  | T1 | 56 | 1 | Ref. | . | . | 29 | 1 | Ref. | . | . |
|  | T2 | 47 | 1.45 | 0.88-2.39 | 0.1419 | 0.0037 | 27 | 0.85 | 0.43-1.68 | 0.6487 | 0.7234 |
|  | T3 | 59 | 2.33 | 1.31-4.15 | 0.0039 | . | 37 | 1.16 | 0.50-2.71 | 0.7309 | . |
| T-2/HT-2 toxins | Per 1 SD increase | 162 | 1.32 | 1.10-1.58 | 0.0033 | . | 93 | 0.95 | 0.71-1.29 | 0.7558 | . |
|  | T1 | 54 | 1 | Ref. | . | . | 30 | 1 | Ref. | . | . |
|  | T2 | 44 | 0.85 | 0.52-1.39 | 0.5199 | 0.1633 | 24 | 0.8 | 0.41-1.54 | 0.4977 | 0.8355 |
|  | T3 | 64 | 1.38 | 0.85-2.23 | 0.1942 | . | 39 | 1.05 | 0.52-2.15 | 0.8873 | . |
| Nivalenol | Per 1 SD increase | 162 | 1.17 | 0.94-1.47 | 0.1591 | . | 93 | 0.8 | 0.57-1.14 | 0.2148 | . |
|  | T1 | 57 | 1 | Ref. | . | . | 31 | 1 | Ref. | . | . |
|  | T2 | 49 | 1.01 | 0.61-1.67 | 0.9640 | 0.0959 | 28 | 0.63 | 0.32-1.24 | 0.1773 | 0.6170 |
|  | T3 | 56 | 1.63 | 0.92-2.89 | 0.0947 | . | 34 | 0.8 | 0.37-1.74 | 0.5777 | . |
| Fumonisins | Per 1 SD increase | 162 | 1.2 | 0.99-1.45 | 0.0666 | . | 93 | 0.64 | 0.40-1.03 | 0.0644 | . |
|  | T1 | 64 | 1 | Ref. | . | . | 33 | 1 | Ref. | . | . |
|  | T2 | 39 | 0.83 | 0.50-1.38 | 0.4701 | 0.1996 | 35 | 1.06 | 0.58-1.97 | 0.8413 | 0.4689 |
|  | T3 | 59 | 1.39 | 0.82-2.35 | 0.2261 | . | 25 | 0.71 | 0.30-1.66 | 0.4261 | . |
| Diacetoxyscirpenol | Per 1 SD increase | 162 | 0.96 | 0.82-1.11 | 0.5501 | . | 93 | 1.17 | 0.80-1.72 | 0.4058 | . |
|  | T1 | 76 | 1 | Ref. | . | . | 33 | 1 | Ref. | . | . |
|  | T2 | 45 | 0.99 | 0.62-1.58 | 0.9659 | 0.9353 | 31 | 1 | 0.53-1.89 | 0.9991 | 0.9313 |
|  | T3 | 41 | 0.98 | 0.55-1.74 | 0.9349 | . | 29 | 0.96 | 0.43-2.17 | 0.9295 | . |
| Zearalenone & derivatives | Per 1 SD increase | 162 | 0.95 | 0.73-1.24 | 0.6968 | . | 93 | 1.14 | 0.85-1.52 | 0.3897 | . |
|  | T1 | 60 | 1 | Ref. | . | . | 44 | 1 | Ref. | . | . |
|  | T2 | 51 | 0.88 | 0.54-1.43 | 0.6035 | 0.9391 | 30 | 0.96 | 0.51-1.78 | 0.8877 | 0.0715 |
|  | T3 | 51 | 1.02 | 0.57-1.83 | 0.9356 | . | 19 | 0.4 | 0.16-1.00 | 0.0502 | . |
| Fusarium Toxins | Per 1 SD increase | 162 | 1.17 | 0.96-1.42 | 0.1184 | . | 93 | 0.86 | 0.55-1.36 | 0.5279 | . |
|  | T1 | 61 | 1 | Ref. | . | . | 28 | 1 | Ref. | . | . |
|  | T2 | 43 | 1.07 | 0.64-1.77 | 0.8008 | 0.0314 | 37 | 1.29 | 0.68-2.45 | 0.4291 | 0.5382 |
|  | T3 | 58 | 1.83 | 1.04-3.20 | 0.0348 | . | 28 | 0.71 | 0.28-1.79 | 0.4715 | . |
| Fusarenon X | Per 1 SD increase | 162 | 1.3 | 1.02-1.66 | 0.0360 | . | 93 | 0.95 | 0.69-1.32 | 0.7771 | . |
|  | 1 | 52 | 1 | Ref. | . | . | 28 | 1 | Ref. | . | . |
|  | T2 | 54 | 1.23 | 0.76-1.98 | 0.4048 | 0.0864 | 25 | 0.82 | 0.42-1.60 | 0.5674 | 0.8325 |
|  | T3 | 56 | 1.64 | 0.93-2.88 | 0.0864 | . | 40 | 1.08 | 0.50-2.35 | 0.8440 | . |
| Sterigmatocystin | Per 1 SD increase | 162 | 1 | 0.61-1.65 | 0.9895 | . | 93 | 0.58 | 0.33-1.03 | 0.0612 | . |
|  | 1 | 70 | 1 | Ref. | . | . | 41 | 1 | Ref. | . | . |
|  | T2 | 56 | 1.04 | 0.69-1.57 | 0.8605 | 0.7414 | 28 | 1 | 0.59-1.70 | 0.9933 | 0.0223 |
|  | T3 | 36 | 0.89 | 0.53-1.52 | 0.6775 | . | 24 | 0.31 | 0.13-0.75 | 0.0094 | . |
| Moniliformine | Per 1 SD increase | 162 | 1.04 | 0.87-1.25 | 0.6694 | . | 93 | 0.81 | 0.47-1.37 | 0.4263 | . |
|  | T1 | 65 | 1 | Ref. | . | . | 37 | 1 | Ref. | . | . |
|  | T2 | 57 | 0.81 | 0.52-1.24 | 0.3293 | 0.2993 | 24 | 0.87 | 0.48-1.55 | 0.6290 | 0.0610 |
|  | T3 | 40 | 0.78 | 0.46-1.31 | 0.3394 | . | 32 | 0.51 | 0.26-1.01 | 0.0539 | . |
| Alternaria toxins | Per 1 SD increase | 162 | 1.31 | 0.99-1.72 | 0.0547 | . | 93 | 0.96 | 0.67-1.39 | 0.8276 | . |
|  | T1 | 49 | 1 | Ref. | . | . | 20 | 1 | Ref. | . | . |
|  | T2 | 63 | 1.71 | 1.06-2.77 | 0.0289 | 0.0264 | 35 | 1.28 | 0.61-2.66 | 0.5096 | 0.9925 |
|  | T3 | 50 | 2 | 1.07-3.72 | 0.0294 | . | 38 | 1.07 | 0.45-2.57 | 0.8798 | . |
| Citrinin | Per 1 SD increase | 162 | 1 | 0.79-1.28 | 0.9675 | . | 93 | 0.57 | 0.38-0.87 | 0.0083 | . |
|  | T1 | 62 | 1 | Ref. | . | . | 37 | 1 | Ref. | . | . |
|  | T2 | 60 | 0.87 | 0.56-1.35 | 0.5276 | 0.1343 | 26 | 0.94 | 0.54-1.66 | 0.8414 | 0.0391 |
|  | T3 | 40 | 0.68 | 0.41-1.13 | 0.1325 | . | 30 | 0.45 | 0.22-0.92 | 0.0296 | . |
| Enniatins | Per 1 SD increase | 162 | 1 | 0.73-1.35 | 0.9784 | . | 93 | 0.97 | 0.66-1.42 | 0.8752 | . |
|  | T1 | 60 | 1 | Ref. | . | . | 23 | 1 | Ref. | . | . |
|  | T2 | 50 | 0.88 | 0.54-1.43 | 0.6148 | 0.9761 | 30 | 1.33 | 0.67-2.62 | 0.4175 | 0.7892 |
|  | T3 | 52 | 1.05 | 0.51-2.14 | 0.8966 | . | 40 | 1.14 | 0.48-2.68 | 0.7678 | . |
| Sum of Mycotoxins | Per 1 SD increase | 162 | 1.27 | 1.00-1.61 | 0.0498 | . | 93 | 0.9 | 0.58-1.37 | 0.6129 | . |
|  | T1 | 53 | 1 | Ref. | . | . | 25 | 1 | Ref. | . | . |
|  | T2 | 55 | 1.4 | 0.85-2.31 | 0.1883 | 0.0411 | 32 | 1.41 | 0.72-2.79 | 0.3183 | 0.7158 |
|  | T3 | 54 | 1.88 | 1.03-3.45 | 0.0412 | . | 36 | 1.2 | 0.48-2.96 | 0.6995 | . |
| Sum of Mycotoxins, using z-scores | Per 1 SD increase | 162 | 1.19 | 0.91-1.55 | 0.2111 | . | 93 | 0.85 | 0.57-1.26 | 0.4250 | . |
|  | T1 | 59 | 1 | Ref. | . | . | 27 | 1 | Ref. | . | . |
|  | T2 | 52 | 1.08 | 0.66-1.78 | 0.7509 | 0.1482 | 34 | 1.18 | 0.62-2.23 | 0.6210 | 0.3982 |
|  | T3 | 51 | 1.59 | 0.86-2.94 | 0.1420 | . | 32 | 0.69 | 0.29-1.63 | 0.3953 | . |

T1; Tertile 1, T2; Tertile 2, T3; Tertile 3, HCC; Hepatocellular carcinom

(*) Fully adjusted model: Energy intake, BMI, Alcohol at recruitment & lifetime alcohol intake, Physical activity index, Smoking status, Education and Diabetes and Coffee consumption

*Mycotoxins for which only insignificant values have been detected are written in Italic font (Citrinin, Diacetoxyscirpenol, Fusarenon X, Sterigmatocystin)*
