# Supplementary material for: Associations between dietary mycotoxins exposures and risk of hepatocellular carcinoma in a European cohort
Source: PLoS One. 2024 Dec 16;19(12):e0315561. doi: 10.1371/journal.pone.0315561 (PMC11649147; doi:10.1371/journal.pone.0315561)
Supplement: S10 Table — (DOCX) [file pone.0315561.s010.docx]

**S10 Table*.*** **Hazard ratios (HR) and their 95 % confidence intervals (CI) for the associations between mycotoxin exposures and i****ntrahepatic biliary tract cancer risk using a fully adjusted model*.**

|  | **MB (middle bound)** | **IBD BW** | | | | |
| --- | --- | --- | --- | --- | --- | --- |
|  | **Mycotoxins μg/BW*day** | **Cases 88** | **HR** | **95% CI** | **Probability Chi Square** | **Trend Test** |
| Ergot alkaloids | Per 1 SD increase |  | 0.96 | 0.66-1.39 | 0.8242 | . |
|  | Τ1 | 40 | 1 | Ref. | . | . |
|  | Τ2 | 25 | 0.69 | 0.33-1.46 | 0.3290 | 0.2601 |
|  | Τ3 | 23 | 0.58 | 0.23-1.50 | 0.2605 | . |
|  |  |  |  |  |  |  |
| Ochratoxins | Per 1 SD increase |  | 1.22 | 1.03-1.45 | 0.0204 | . |
|  | Τ1 | 33 | 1 | Ref. | . | . |
|  | Τ2 | 24 | 0.87 | 0.44-1.72 | 0.6848 | 0.4242 |
|  | Τ3 | 31 | 1.29 | 0.63-2.66 | 0.4873 | . |
|  |  |  |  |  |  |  |
| Aflatoxins | Per 1 SD increase |  | 1.16 | 0.87-1.55 | 0.3013 | . |
|  | Τ1 | 28 | 1 | Ref. | . | . |
|  | Τ2 | 28 | 1.23 | 0.62-2.44 | 0.5549 | 0.7428 |
|  | Τ3 | 32 | 1.16 | 0.52-2.62 | 0.7157 | . |
|  |  |  |  |  |  |  |
| Patulin | Per 1 SD increase |  | 1.17 | 0.95-1.45 | 0.1385 | . |
|  | Τ1 | 29 | 1 | Ref. | . | . |
|  | Τ2 | 30 | 1.29 | 0.67-2.48 | 0.4389 | 0.2350 |
|  | Τ3 | 29 | 1.51 | 0.77-2.95 | 0.2322 | . |
|  |  |  |  |  |  |  |
| Deoxynivalenol and derivatives | Per 1 SD increase |  | 1.08 | 0.75-1.54 | 0.6876 | . |
|  | Τ1 | 34 | 1 | Ref. | . | . |
|  | Τ2 | 33 | 1.39 | 0.72-2.68 | 0.3302 | 0.5760 |
|  | Τ3 | 21 | 1.27 | 0.54-2.98 | 0.5865 | . |
|  |  |  |  |  |  |  |
| T-2/HT-2 toxins | Per 1 SD increase |  | 1.14 | 1.03 -1.04 | 0.2696 | . |
|  | Τ1 | 29 | 1 | Ref. | . | . |
|  | Τ2 | 26 | 1.02 | 0.51-2.04 | 0.9659 | 0.0857 |
|  | Τ3 | 33 | 1.76 | 0.88-3.52 | 0.1078 | . |
|  |  |  |  |  |  |  |
| Nivalenol | Per 1 SD increase |  | 1.08 | 0.90-1.30 | 0.4442 | . |
|  | Τ1 | 33 | 1 | Ref. | . | . |
|  | Τ2 | 29 | 1.43 | 0.73-2.81 | 0.2927 | 0.2727 |
|  | Τ3 | 26 | 1.55 | 0.71-3.37 | 0.2673 | . |
|  |  |  |  |  |  |  |
| Fumonisins | Per 1 SD increase |  | 0.63 | 0.05-8.82 | 0.7317 | . |
|  | Τ1 | 40 | 1 | Ref. | . | . |
|  | Τ2 | 22 | 0.77 | 0.39-1.51 | 0.4453 | 0.8632 |
|  | Τ3 | 26 | 1.06 | 0.49-2.27 | 0.8851 | . |
|  |  |  |  |  |  |  |
| *Diacetoxyscirpenol* | Per 1 SD increase |  | *0.98* | *0.86-1.12* | *0.8807* | *.* |
|  | *Τ1* | *37* | *1* | *Ref.* | *.* | *.* |
|  | *Τ2* | *26* | *0.69* | *0.36-1.35* | *0.2832* | *0.9713* |
|  | *Τ3* | *25* | *0.99* | *0.46-2.12* | *0.9780* | *.* |
|  |  |  |  |  |  |  |
| Zearalenone & derivatives | Per 1 SD increase |  | 1.00 | 0.82-1.22 | 0.1448 | . |
|  | Τ1 | 37 | 1 | Ref. | . | . |
|  | Τ2 | 25 | 1.24 | 0.64-2.39 | 0.5310 | 0.2270 |
|  | Τ3 | 26 | 1.63 | 0.74-3.62 | 0.2259 | . |
|  |  |  |  |  |  |  |
| Fusarium Toxins | Per 1 SD increase |  | 1.12 | 0.94-1.33 | 0.8151 | . |
|  | Τ1 | 35 | 1 | Ref. | . | . |
|  | Τ2 | 29 | 1.09 | 0.56-2.13 | 0.8001 | 0.5537 |
|  | Τ3 | 24 | 1.29 | 0.55-3.00 | 0.5543 | . |
|  |  |  |  |  |  |  |
| *Fusarenon X* | Per 1 SD increase |  | *1.19* | *0.98-1.44* | *0.8024* | *.* |
|  | *Τ1* | *34* | *1* | *Ref.* | *.* | *.* |
|  | *Τ2* | *33* | *1.18* | *0.63-2.20* | *0.6059* | *0.7899* |
|  | *Τ3* | *21* | *0.88* | *0.40-1.96* | *0.7559* | *.* |
|  |  |  |  |  |  |  |
| *Sterigmatocystins* | Per 1 SD increase |  | *0.79* | *0.55-1.13* | *0.4166* | *.* |
|  | *Τ1* | *32* | *1* | *Ref.* | *.* | *.* |
|  | *Τ2* | *31* | *1.05* | *0.59-1.86* | *0.8744* | *0.9263* |
|  | *Τ3* | *25* | *0.96* | *0.48-1.90* | *0.8976* | *.* |
|  |  |  |  |  |  |  |
| Moniliformine | Per 1 SD increase |  | 0.98 | 0.80-1.21 | 0.7111 | . |
|  | Τ1 | 33 | 1 | Ref. | . | . |
|  | Τ2 | 32 | 0.77 | 0.41-1.45 | 0.4194 | 0.2012 |
|  | Τ3 | 23 | 0.66 | 0.35-1.26 | 0.2100 | . |
|  |  |  |  |  |  |  |
| Alternaria toxins | Per 1 SD increase |  | 1.18 | 0.95-1.47 | 0.4306 | . |
|  | Τ1 | 31 | 1 | Ref. | . | . |
|  | Τ2 | 33 | 1.03 | 0.53-2.00 | 0.9264 | 0.5589 |
|  | Τ3 | 24 | 0.79 | 0.34-1.83 | 0.5770 | . |
|  |  |  |  |  |  |  |
| *Citrinin* | Per 1 SD increase |  | *0.82* | *0.66-1.01.* | *0.0860* | *.* |
|  | *Τ1* | *31* | *1* | *Ref.* | *.* | *.* |
|  | *Τ2* | *38* | *1.04* | *0.58-1.88* | *0.8973* | *0.3370* |
|  | *Τ3* | *19* | *0.71* | *0.37-1.39* | *0.3191* | *.* |
|  |  |  |  |  |  |  |
| Enniatins | Per 1 SD increase |  | 0.99 | 0.78-1.26 | 0.1215 | . |
|  | Τ1 | 37 | 1 | Ref. | . | . |
|  | Τ2 | 28 | 0.73 | 0.38-1.41 | 0.3434 | 0.1853 |
|  | Τ3 | 23 | 0.55 | 0.23-1.35 | 0.1944 | . |
|  |  |  |  |  |  |  |
| Sum of Mycotoxins | Per 1 SD increase |  | 01.17 | 0.96-1.43 | 0.8214 | . |
|  | Τ1 | 37 | 1 | Ref. | . | . |
|  | Τ2 | 27 | 1.03 | 0.52-2.01 | 0.9373 | 0.8106 |
|  | Τ3 | 24 | 1.11 | 0.47-2.64 | 0.8106 | . |
|  |  |  |  |  |  |  |
| Sum of Mycotoxins, using z-scores | Per 1 SD increase |  | 1.05 | 0.85-1.31 | 0.9605 | . |
|  | Τ1 | 39 | 1 | Ref. | . | . |
|  | Τ2 | 20 | 0.66 | 0.33-1.34 | 0.2480 | 0.7703 |
|  | Τ3 | 29 | 1.11 | 0.48-2.57 | 0.7984 | . |

T1; Tertile 1, T2; Tertile 2, T3; Tertile 3, IBD; intrahepatic biliary tract, BW; body weight

(*) Fully adjusted model: Energy intake, BMI, Alcohol at recruitment & lifetime alcohol intake, Physical activity index, Smoking status, Education and Diabetes and Coffee consumption.

Mycotoxins for which only insignificant values have been detected are written in Italic font (Citrinin, Diacetoxyscirpenol, Fusarenon X, Sterigmatocystin).
