# Supplementary material for: Associations between dietary mycotoxins exposures and risk of hepatocellular carcinoma in a European cohort
Source: PLoS One. 2024 Dec 16;19(12):e0315561. doi: 10.1371/journal.pone.0315561 (PMC11649147; doi:10.1371/journal.pone.0315561)
Supplement: S11 Table — (DOCX) [file pone.0315561.s011.docx]

**S11 Table. Hazard ratios (HR) and their 95 % confidence intervals (CI) for the associations between mycotoxin exposures and extra-hepatic biliary tract cancer risk using a fully adjusted model*.**

|  | **MB (middle bound)** | **EBD BW** | | | | |
| --- | --- | --- | --- | --- | --- | --- |
|  | **Mycotoxins μg*BW/day** | **Cases 85** | **HR** | **95% CI** | **Probability Chi Square test** | **Trend Test** |
| Ergot alkaloids | Per 1 SD increase |  | 0.74 | 0.51-1.09 | 0.1291 | . |
|  | Τ1 | 20 | 1 | Ref. | . | . |
|  | Τ2 | 33 | 1.43 | 0.65-3.18 | 0.3759 | 0.5807 |
|  | Τ3 | 32 | 1.37 | 0.53-3.53 | 0.5197 | . |
|  |  |  |  |  |  |  |
| Ochratoxins | Per 1 SD increase |  | 0.93 | 0.65-1.32 | 0.6893 | . |
|  | Τ1 | 32 | 1 | Ref. | . | . |
|  | Τ2 | 22 | 0.54 | 0.27-1.07 | 0.0763 | 0.6296 |
|  | Τ3 | 31 | 0.82 | 0.39-1.69 | 0.5860 | . |
|  |  |  |  |  |  |  |
| Aflatoxins | Per 1 SD increase |  | 0.99 | 0.67-1.47 | 0.9587 | . |
|  | Τ1 | 34 | 1 | Ref. | . | . |
|  | Τ2 | 27 | 0.74 | 0.39-1.40 | 0.3596 | 0.4766 |
|  | Τ3 | 24 | 0.76 | 0.35-1.69 | 0.5073 | . |
|  |  |  |  |  |  |  |
| Patulin | Per 1 SD increase |  | 0.75 | 0.53-1.08 | 0.1208 | . |
|  | Τ1 | 38 | 1 | Ref. | . | . |
|  | Τ2 | 24 | 0.56 | 0.31-1.03 | 0.0610 | 0.0509 |
|  | Τ3 | 23 | 0.54 | 0.28-1.04 | 0.0662 | . |
|  |  |  |  |  |  |  |
| Deoxynivalenol and derivatives | Per 1 SD increase |  | 0.85 | 0.57-1.25 | 0.4048 | . |
|  | Τ1 | 30 | 1 | Ref. | . | . |
|  | Τ2 | 32 | 1.53 | 0.77-3.04 | 0.2258 | 0.5836 |
|  | Τ3 | 23 | 1.28 | 0.53-3.11 | 0.5810 | . |
|  |  |  |  |  |  |  |
| T-2/HT-2 toxins | Per 1 SD increase |  | 0.72 | 0.49-1.06 | 0.0928 | . |
|  | Τ1 | 37 | 1 | Ref. | . | . |
|  | Τ2 | 24 | 0.61 | 0.31-1.17 | 0.1337 | 0.2929 |
|  | Τ3 | 24 | 0.69 | 0.34-1.40 | 0.3031 | . |
|  |  |  |  |  |  |  |
| Nivalenol | Per 1 SD increase |  | 0.73 | 0.50-1.08 | 0.1151 | . |
|  | Τ1 | 32 | 1 | Ref. | . | . |
|  | Τ2 | 30 | 0.75 | 0.40-1.42 | 0.3783 | 0.0743 |
|  | Τ3 | 23 | 0.47 | 0.21-1.07 | 0.0733 | . |
|  |  |  |  |  |  |  |
| Fumonisins | Per 1 SD increase |  | 0.81 | 0.53-1.23 | 0.3249 | . |
|  | Τ1 | 32 | 1 | Ref. | . | . |
|  | Τ2 | 30 | 1.09 | 0.57-2.08 | 0.7994 | 0.8622 |
|  | Τ3 | 23 | 1.07 | 0.48-2.39 | 0.8644 | . |
|  |  |  |  |  |  |  |
| *Diacetoxyscirpenol* | Per 1 SD increase |  | *0.94* | *0.69-1.28* | *0.7027* | *.* |
|  | *Τ1* | *33* | *1* | *Ref.* | *.* | *.* |
|  | *Τ2* | *26* | *0.97* | *0.50-1.87* | *0.9160* | *0.6570* |
|  | *Τ3* | *26* | *1.2* | *0.54-2.68* | *0.6489* | *.* |
|  |  |  |  |  |  |  |
| Zearalenone & derivatives | Per 1 SD increase |  | 0.66 | 0.44-1.01 | 0.0530 | . |
|  | Τ1 | 34 | 1 | Ref. | . | . |
|  | Τ2 | 28 | 0.88 | 0.46-1.67 | 0.6890 | 0.1816 |
|  | Τ3 | 23 | 0.59 | 0.27-1.28 | 0.1805 | . |
|  |  |  |  |  |  |  |
| Fusarium Toxins | Per 1 SD increase |  | 0.75 | 0.49-1.15 | 0.1897 | . |
|  | Τ1 | 32 | 1 | Ref. | . | . |
|  | Τ2 | 35 | 1.66 | 0.86-3.21 | 0.1303 | 0.9440 |
|  | Τ3 | 18 | 0.94 | 0.38-2.32 | 0.8995 | . |
|  |  |  |  |  |  |  |
| *Fusarenon X* | Per 1 SD increase |  | *0.90* | *0.62-1.32* | *0.5997* | *.* |
|  | *Τ1* | *28* | *1* | *Ref.* | *.* | *.* |
|  | *Τ2* | *32* | *1.55* | *0.80-3.00* | *0.1951* | *0.6425* |
|  | *Τ3* | *25* | *1.22* | *0.53-2.78* | *0.6428* | *.* |
|  |  |  |  |  |  |  |
| *Sterigmatocystins* | Per 1 SD increase |  | *1.12* | *0.71-1.77* | *0.6198* | *.* |
|  | *Τ1* | *35* | *1* | *Ref.* | *.* | *.* |
|  | *Τ2* | *27* | *0.69* | *0.38-1.27* | *0.2376* | *0.4997* |
|  | *Τ3* | *23* | *0.84* | *0.42-1.68* | *0.6214* | *.* |
|  |  |  |  |  |  |  |
| Moniliformine | Per 1 SD increase |  | 1.11 | 0.94-1.32 | 0.2136 | . |
|  | Τ1 | 30 | 1 | Ref. | . | . |
|  | Τ2 | 29 | 0.87 | 0.47-1.62 | 0.6614 | 0.9573 |
|  | Τ3 | 26 | 1.03 | 0.52-2.05 | 0.9278 | . |
|  |  |  |  |  |  |  |
| Alternaria toxins | Per 1 SD increase |  | 0.57 | 0.37-0.87 | 0.0101 | . |
|  | Τ1 | 33 | 1 | Ref. | . | . |
|  | Τ2 | 27 | 0.61 | 0.31-1.20 | 0.1536 | 0.0218 |
|  | Τ3 | 25 | 0.37 | 0.16-0.87 | 0.0220 | . |
|  |  |  |  |  |  |  |
| *Citrinin* | Per 1 SD increase |  | *0.97* | *0.73-1.29* | *0.8411* | *.* |
|  | *Τ1* | *31* | *1* | *Ref.* | *.* | *.* |
|  | *Τ2* | *26* | *0.84* | *0.45-1.59* | *0.5964* | *0.9323* |
|  | *Τ3* | *28* | *0.97* | *0.50-1.91* | *0.9367* | *.* |
|  |  |  |  |  |  |  |
| Enniatins | Per 1 SD increase |  | 0.83 | 0.55-1.24 | 0.3623 | . |
|  | Τ1 | 26 | 1 | Ref. | . | . |
|  | Τ2 | 29 | 0.95 | 0.50-1.84 | 0.8887 | 0.7231 |
|  | Τ3 | 30 | 0.85 | 0.35-2.06 | 0.7141 | . |
|  |  |  |  |  |  |  |
| Sum of Mycotoxins | Per 1 SD increase |  | 0.65 | 0.42-1.00 | 0.0520 | . |
|  | Τ1 | 32 | 1 | Ref. | . | . |
|  | Τ2 | 29 | 1.07 | 0.55-2.09 | 0.8402 | 0.5181 |
|  | Τ3 | 24 | 0.74 | 0.30-1.81 | 0.5114 | . |
|  |  |  |  |  |  |  |
| Sum of Mycotoxins, using z-scores | Per 1 SD increase |  | 0.62 | 0.41-0.95 | 0.0288 | . |
|  | Τ1 | 30 | 1 | Ref. | . | . |
|  | Τ2 | 32 | 0.93 | 0.49-1.80 | 0.8387 | 0.0942 |
|  | Τ3 | 23 | 0.46 | 0.19-1.12 | 0.0880 | . |

*T1; Tertile 1, T2; Tertile 2, T3; Tertile 3, EBD; extra-hepatic biliary tract, BW; body weight*

*(*) Fully adjusted model: Energy intake, BMI, Alcohol at recruitment & lifetime alcohol intake, Physical activity index, Smoking status, Education and Diabetes and Coffee consumption.*

*Mycotoxins for which only insignificant values have been detected are written in Italic font (Citrinin, Diacetoxyscirpenol, Fusarenon X, Sterigmatocystin).*
