# Supplementary material for: Associations between dietary mycotoxins exposures and risk of hepatocellular carcinoma in a European cohort
Source: PLoS One. 2024 Dec 16;19(12):e0315561. doi: 10.1371/journal.pone.0315561 (PMC11649147; doi:10.1371/journal.pone.0315561)
Supplement: S13 Table — (DOCX) [file pone.0315561.s013.docx]

**S13 Table. Sources of mycotoxins: Percentage (%) contributions from the main EPIC food groups (middle bound values).**

|  | | | Mycotoxin groups | | | | | | | | | | | Individual Mycotoxins | | |
| --- | --- | --- | --- | --- | --- | --- | --- | --- | --- | --- | --- | --- | --- | --- | --- | --- |
|  |  |  | Sum of Mycotoxins | Ergot alkaloids | Ochratoxins | Aflatoxins | Deoxynivalenol and derivative | T-2/HT-2 toxins | Fumonisins | Zearalenone & derivatives | Alternaria toxins | Enniatins | Moniliformine | | Patulin |  |
| **Epic Food Classification** | Middle Bound  N (2% exclusion) | | % | % | % | % | % | % | % | % | % | % | % | | % |  |
| Potatoes and other tubers | | 499547 | 1.03 | 0.00 | 0.00 | 0.00 | 0.00 | 0.01 | 0.00 | 0.01 | 5.40 | 0.01 | 0.00 | | 0.00 |  |
| Vegetables | | 504155 | 20.30 | 0.00 | 41.54 | 95.88 | 10.70 | 4.62 | 11.04 | 5.62 | 11.23 | 0.00 | 0.00 | | 9.48 |  |
| Legumes | | 395089 | 0.58 | 0.00 | 0.12 | 0.34 | 0.69 | 1.50 | 0.00 | 0.31 | 0.92 | 0.00 | 0.00 | | 0.00 |  |
| Fruits, nuts and seeds | | 502312 | 11.70 | 0.00 | 10.05 | 0.71 | 14.61 | 41.39 | 0.01 | 6.50 | 19.85 | 0.01 | 0.00 | | 34.54 |  |
| Dairy products | | 503526 | 2.85 | 4.02 | 0.25 | 0.06 | 4.52 | 0.13 | 0.04 | 20.64 | 0.11 | 0.05 | 1.23 | | 3.30 |  |
| Cereal and cereal products | | 504128 | 39.12 | 75.43 | 12.81 | 0.31 | 44.21 | 30.13 | 34.34 | 26.53 | 43.34 | 91.00 | 79.37 | | 0.00 |  |
| Meat and meat products | | 503168 | 0.64 | 0.00 | 6.10 | 0.36 | 1.29 | 0.00 | 0.00 | 2.11 | 0.00 | 0.00 | 0.00 | | 0.00 |  |
| Fish and shellfish | | 479036 | 0.05 | 0.00 | 1.79 | 0.12 | 0.07 | 0.28 | 0.00 | 0.24 | 0.00 | 0.00 | 0.00 | | 0.08 |  |
| Egg and egg products | | 495665 | 0.04 | 0.00 | 0.00 | 0.00 | 0.00 | 0.00 | 0.00 | 1.11 | 0.00 | 0.00 | 0.00 | | 0.00 |  |
| Fat | | 504190 | 2.48 | 0.00 | 1.65 | 0.18 | 1.78 | 0.31 | 0.00 | 13.61 | 1.50 | 3.19 | 0.00 | | 0.00 |  |
| Sugar and confectionary | | 492622 | 2.31 | 0.00 | 5.71 | 0.17 | 2.53 | 6.55 | 0.02 | 1.46 | 3.68 | 0.00 | 0.00 | | 4.67 |  |
| Cakes and biscuits | | 480647 | 10.13 | 20.23 | 4.69 | 0.35 | 7.35 | 5.48 | 36.94 | 5.88 | 7.64 | 2.43 | 18.11 | | 1.65 |  |
| Non-alcoholic beverages | | 502437 | 1.47 | 0.00 | 10.12 | 0.52 | 0.79 | 0.29 | 0.54 | 0.54 | 2.14 | 0.00 | 0.00 | | 37.38 |  |
| Alcoholic beverages | | 442798 | 5.35 | 0.00 | 2.47 | 0.01 | 9.69 | 5.90 | 15.20 | 12.64 | 3.82 | 0.00 | 0.00 | | 8.10 |  |
| Condiments and sauces | | 491258 | 0.48 | 0.02 | 2.38 | 0.04 | 0.92 | 2.95 | 0.01 | 2.03 | 0.20 | 0.35 | 0.09 | | 0.26 |  |
| Soups, bouillons | | 408790 | 0.50 | 0.30 | 0.23 | 0.89 | 0.49 | 0.26 | 1.86 | 0.61 | 0.08 | 0.13 | 1.21 | | 0.54 |  |
| Miscellaneous | | 209789 | 0.96 | 0.00 | 0.08 | 0.05 | 0.37 | 0.19 | 0.01 | 0.15 | 0.11 | 2.83 | 0.00 | | 0.00 |  |
